# Differential Expression of Non-Coding RNA Signatures in Thyroid Cancer between Two Ethnic Groups

**DOI:** 10.3390/curroncol28050309

**Published:** 2021-09-19

**Authors:** Kristiana Rood, Khodeza Begum, Hanmin Wang, Yan C. Wangworawat, Ryan Davis, Celina R. Yamauchi, Mia C. Perez, Alfred A. Simental, Ria T. Laxa, Charles Wang, Sourav Roy, Salma Khan

**Affiliations:** 1 Division of Biochemistry, Loma Linda University School of Medicine, Loma Linda, CA 92350, USA; krood@students.llu.edu (K.R.); hwang1@students.llu.edu (H.W.); rsdavis@students.llu.edu (R.D.); romi.yamauchi@gmail.com (C.R.Y.); rtlaxa@dons.usfca.edu (R.T.L.); 2 Center for Health Disparities & Molecular Medicine, Loma Linda University School of Medicine, Loma Linda, CA 92350, USA; 3 Department of Biological Sciences, University of Texas El Paso, El Paso, TX 79968, USA; kbegum@utep.edu; 4 Border Biomedical Research Center, University of Texas El Paso, El Paso, TX 79968, USA; 5 Department of Pathology & Human Anatomy, Loma Linda University School of Medicine, Loma Linda, CA 92350, USA; mousechenyan@gmail.com (Y.C.W.); miperez@llu.edu (M.C.P.); 6 Division of Otolaryngology, Loma Linda University School of Medicine, Loma Linda, CA 92350, USA; asimenta@llu.edu; 7 Center for Genomics, Loma Linda University School of Medicine, Loma Linda, CA 92350, USA; cwang@llu.edu; 8 Department of Internal Medicine, Loma Linda University School of Medicine, Loma Linda, CA 92354, USA

**Keywords:** miRNA, thyroid cancer, health disparities, prognosis

## Abstract

Filipino Americans show higher thyroid cancer recurrence rates compared to European Americans. Although they are likely to die of this malignancy, the molecular mechanism has not yet been determined. Recent studies demonstrated that small non-coding RNAs could be utilized to assess thyroid cancer prognosis in tumor models. The goal of this study is to determine whether microRNA (miRNA) signatures are differentially expressed in thyroid cancer in two different ethnic groups. We also determined whether these miRNA signatures are related to cancer staging. This is a retrospective study of archival samples from patients with thyroid cancer (both sexes) in the pathology division from the last ten years at Loma Linda University School of Medicine, California. Deidentified patient demographics were extracted from the patient chart. Discarded formalin-fixed paraffin-embedded tissues were collected post-surgeries. We determined the differential expressions of microRNA in archival samples from Filipino Americans compared to European Americans using the state-of-the-art technique, HiSeq4000. By ingenuity pathway analysis, we determined miRNA targets and the pathways that those targets are involved in. We validated their expressions by real-time quantitative PCR and correlated them with the clinicopathological status in a larger cohort of miRNA samples from both ethnicities. We identified the differentially upregulated/downregulated miRNA clusters in Filipino Americans compared to European Americans. Some of these miRNA clusters are known to target genes that are linked to cancer invasion and metastasis. In univariate analysis, ethnicity and tumor staging were significant factors predicting miR-4633-5p upregulation. When including these factors in a multivariate logistic regression model, ethnicity and tumor staging remained significant independent predictors of miRNA upregulation, whereas the interaction of ethnicity and tumor staging was not significant. In contrast, ethnicity remained an independent predictor of significantly downregulated miR-491-5p and let-7 family. We provide evidence that Filipino Americans showed differentially expressed tumor-tissue-derived microRNA clusters. The functional implications of these miRNAs are under investigation.

## 1. Introduction

Thyroid cancer (TC) is the most common type of endocrine malignancy, and the incidence of TC has increased in the last few decades [1,2,3,4]. A higher rate of TC has been identified in different racial/ethnic populations in several studies [5,6,7]. Monitoring cancer trends is one of the most critical functions of a cancer surveillance system [8]. The National Cancer Institute also recognizes Asians as one of the most at-risk ethnicities for TC [9]. In particular, Filipinos have a notably higher risk of developing TC compared to other races/ethnicities [10,11,12]. The annual incidence of TC among Filipino men and women in the United States is estimated at 7.3 and 17.3 cases per 100,000, respectively. Papillary thyroid cancer (PTC) is the most common TC subtype. Although the prognosis of PTC is good, on average, 50% of the patients with PTC are characterized by metastasis to the cervical lymph nodes (LNs). Data showed that Filipino women have a higher incidence rate with a peak age range from 45–54. In Filipino American women, a higher rate of recurrence was reported, especially in premenopausal women. The long-term survival rate was also lower in those females compared to any other ethnic group [7,8,12,13,14,15,16]. Recently, it has been found that the recurrence rate is also higher in Filipino Americans [7,10,12,15,16]. Therefore, identifying biomarkers for the prognosis of PTC in Filipino Americans is one of the most important issues in TC health disparities for monitoring cancer progression. 

Recent studies have identified the link between miRNA expression and cancer, where significant changes in expression have been detected in malignant cells when compared to benign cells [17]. Many cancers, including chronic lymphocytic leukemia, colon cancer, glioblastoma, and astrocytoma [18,19], have already been associated with changes in the levels of miRNA expression. As a result, miRNAs can be tested as potential diagnostic or prognostic biomarkers. Therefore, miRNA expression profiling in thyroid cancer has the potential for being used as a diagnostic or prognostic marker. One of the striking features of miRNA is its molecular stability, unlike mRNAs; miRNAs are well-preserved in formalin-fixed paraffin-embedded (FFPE) tissue, which renders miRNAs excellent candidates for biomarkers.

miRNAs are classified as oncogenes or tumor suppressor genes. It is important to know that miRNAs should not always be classified as tumor suppressors or oncogenes; their classification depends on the tissue or cell type in which they are expressed [20]. These genes regulate different carcinogenic processes in various types of malignancies [21]. miRNAs are involved in tumor formation by regulating target genes. Using a large cohort of samples, and great advancement of technologies, miRNAs could be isolated from serum and fresh tumor tissues/archival samples, detected by high throughput HiSeq 4000, and confirmed by RT-qPCR techniques.

The deregulation of miRNA profiles has been used not only as a diagnostic marker but also as a predictive marker in various types of malignancies, including thyroid cancer [22]. In this study, we optimized pure miRNA extraction from FFPE tissues and determined the differentially expressed miRNA clusters by state-of-the-art HiSeq4000 analysis from the archival samples of Filipino Americans compared to European American patients with thyroid cancer. With the help of Ingenuity Pathway Analysis (IPA) software tools, we also determined the pathways that are potentially regulated by these small RNAs. Finally, we validated our data by quantitative real-time PCR (RT-q-PCR) assays from the most relevant miRNAs in cancers (miR-4633-5p, miR-323-3p, miR-6775-3p, miR-4749-3p, miR-323b-3p, miR-491-5p, and let-7 family). Some of them were differentially expressed in thyroid cancer tissue samples and showed a stage-dependent increment of the expression profile.

## 2. Materials and Methods

### 2.1. Tumor Sample Selection

We initially selected 10 patients with thyroid cancer from both Filipino Americans (*n* = 5) and European Americans (*n* = 5) for miRNA-seq. We expanded our pilot study by acquiring more archival thyroid tissues and fresh tissues from both Filipino American (*n* = 50) and European American (*n* = 50) men and women. The samples were obtained from the biorepository as well as the Departments of Pathology at Loma Linda University Medical Center (LLUMC) and Riverside University Health System (RUHS) and included tissues from the years 2004 to 2019. This study consists of de-identified discarded tissue samples from both Filipino American (1st generation Filipinos who have resided in the USA for the last 20 years) and European American (Europeans who have been residing in the USA) patients with thyroid cancer (ethnicity status was recorded according to their self-identified ethnic group), aged 18–80 years, and from both sexes with adequate clinical information and paraffin blocks for immunohistochemistry (IHC).

### 2.2. Histological Examination

We confirmed all pathological diagnoses of samples using established histopathological criteria using routine hematoxylin and eosin (H&E) staining by our expert pathologists. We included papillary thyroid cancer subtypes (PTC) from both ethnicities to maintain genetic uniformity and tissue specificity. Microdissected cancer samples were obtained after being postmarked by pathologists.

### 2.3. Deidentified Patient Demographics

We obtained deidentified patient information, including demographic data (age and sex), tumor size, extrathyroidal extension, nodal status, and distant metastases (for determining disease stage) by the independent review of de-identified charts (pathological tumor, node, metastasis, TNM staging). All samples were obtained under IRB-approved studies according to the university and hospital policies at both LLU and RUHS.

### 2.4. miRNA Isolation, miRNA-Seq Library Preparation, Validation, and HiSeq4000 Sequencing

For purification of miRNA and total RNA from formalin-fixed paraffin-embedded (FFPE) microdissected samples, an H&E section was consulted in each case to guide the area of interest, as described before [23]. We used an all-prep miRNA FFPE kit (QIAGEN, Valencia, CA, USA) and the microdissection was performed after evaluation of the tumor area. After the H&E section, tumor areas were identified by the pathologist, we marked the tumor area by pap-pen, and then the tumor area was resected using blades and collected in 1 mL tube and washed with xylene and 100% ethanol sequentially for cleaning paraffin from the tumor tissues according to the instructions of the kit (QIAGEN). This kit allowed us to effectively purify both miRNA and total RNA within a short period, and via specific lysis and incubation conditions, reverse the formalin crosslinking with high quantity and quality. miRNA was isolated according to the instructions of the kit. The quality and quantity of miRNA were recorded by using Nanodrop (NanoDrop Technologies, Waltham, MA, USA) and the QIA-seq miRNA quantification tool. RNA samples were excluded from further analysis if OD 260/280 < 1.8. We also confirmed RNA quality by Bioanalyzer.

### 2.5. Library Preparation and Validation for Small Non-Coding RNA (sncRNA)-Seq

**Figure 1:**
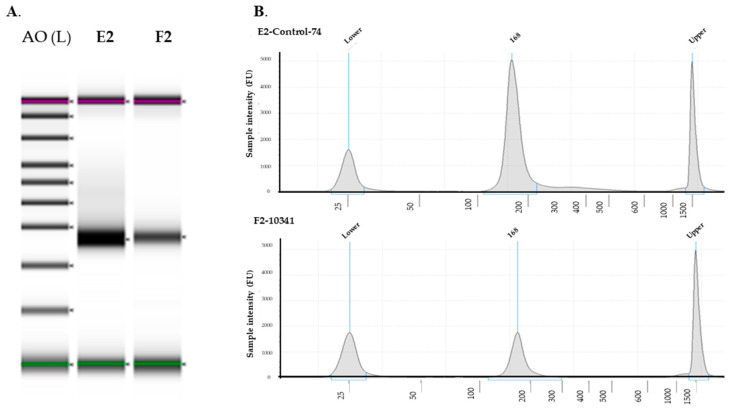
miRNA quality analysis. miRNA was isolated from FFPE tissues from European Americans and Filipino Americans. (**A**) Quality was checked by gel image, (**B**) miRNA QC was performed by RNA screentape. A0, ladder; E2, European cancer tissue; F2, Filipino cancer tissue. QC, quality control.

A library of small RNAs was prepared from the FFPE samples of TC using QIAseq™ miRNA Library Kit (QIAGEN). We also established high-quality libraries with RNA Integrity Numbers (RINs) > 8, and RNA quality was checked by running on agarose gel to visualize 28S and 18S RNA (Figure 1A,B) with a 2:1 ratio. miRNA-seq library kit is a beads-based method, eliminating adapter dimers and unwanted RNA species resulting in high fidelity and the most efficient data. We prepared total RNA miRNA-seq libraries manually following the manufacturer’s protocol with 200 ng total RNA input. In brief, adapters were ligated sequentially to the 3′ and 5′ ends of miRNAs, then cDNA was synthesized with the UMI assignment. After cDNA cleanup, libraries were multiplexed with QIAseq miRNA NGS indexes and amplified by PCR, using the following conditions: 95 °C 15 min; 13 cycles of (95 °C 15 s, 60 °C 30 s, and 72 °C 15 s); 72 °C 2 min. We purified the amplified library with AMPure XP beads, verified with TapeStation 2200 using D1000 Screentae (Agilent). These steps followed the massively parallel sequencing of millions of cDNA molecules from the library. 

### 2.6. Sequencing and Fastq Generation

Illumina HiSeq4000 SR flowcell was clustered on cBot with SR cluster kit from 5 uL of 2 nM template. Sequencing was performed on an Illumina HiSeq 4000 platform using a 75-base single-read protocol. We performed the sequencing on an Illumina HiSeq 4000 using single-read 100-cycle SBS reagents. Base call files (*.bcl) were converted to FASTQ and sorted using bcl2fastq scripts (Illumina), with one miss-matched barcode allowed. The quality of the sequenced reads for each sample was assessed using the FastQC program (http://www.bioinformatics.babraham.ac.uk/projects/fastqc, accessed on 20 July 2016). Reads shorter than 20 bp or with a Phred quality score (Q score) less than 20 were removed. We first converted the raw sequencing data to unique molecular indices (UMI) and then converted the UMI to fold-change value by directly using the Primary QIAseq miRNA Quantification Data Analysis Output Excel file as the Secondary QIAseq miRNA Library Kit Data Analysis input file. Integrated UMIs enabled the quantification of individual miRNA molecules, eliminating PCR and sequence bias. Primary read mapping and differential expression analysis were performed via GeneGlobe Analysis Center (QIAGEN). The secondary QIAGEN miRNA library kit data analysis software analyzed the UMI counts to calculate changes in miRNA expression. It used automated normalization through geNorm and total UMI count normalization methods.

### 2.7. Fold Regulation Comparison and p-Value

Samples were assigned to controls and test groups. The average ratios of normalized UMI counts (or relative miRNA expression) between the control group and each test group were used to calculate the fold change. To identify differential expression, a widely used and accepted cut-off of 2-fold change was used. We analyzed the biological pathways in supplemental attachments using both the Ingenuity Pathway Analysis (IPA) and KEGG map. For the geNorm and total UMI count normalized methods, *p*-values were calculated using Student’s t-test (two-tail distribution and equal variances between two samples) on the replicate normalized UMI counts. Each miRNA in each test group was compared to the control group. The *p*-value was calculated by a parametric, unpaired, two-sample equal variance with two-tailed distribution. Any *p*-value less than 0.05 was indicated in red. Each group (including the control group) contains at least three samples for the software to calculate the *p*-value. 

To identify the top ten miRNAs that were up- or downregulated in the Filipino Americans set when compared to the European Americans set, we used a cut-off of a 2-fold change before sorting them based on highest to lowest fold change (both +ve and −ve). 

#### Ingenuity Pathway Analysis (IPA)

We used the entire set of up- and downregulated miRNAs separately as inputs for the Ingenuity Pathway Analysis (IPA), a web-based software from QIAGEN © 2021–2021, to analyze the relation to diseases, their functions, and to identify the putative targets for these miRNAs. We used the IPA diseases and functions analysis and the target filter analysis tools with default parameters. IPA miRNA Target Filtering allowed us to deduce the genes targeted by miRNAs along with identifying potential relationships between miRNAs and their target genes. Overall, this helped in visualizing which mRNAs are being targeted by a given miRNA.

### 2.8. Quantification of miRs and mRNAs by Reverse-Transcription Quantitative PCR (RT-qPCR)

Quantification of miRNA expression and miRNA-seq data validations were performed by RT-qPCR assay. cDNA was synthesized with 100 ng of total RNA (including miRs) using MystiCq^TM^microRNA cDNA synthesis mix (Sigma-Aldrich, St. Louis, MO, USA). We used an equal number of samples from both ethnicities (Filipino Americans = 50 and European Americans = 50). RT-qPCR for miRNA was performed using iQSYBR^(R)^ Green PCR Supermix (BIO-RAD, Hercules, CA, USA) with specific primers for both miR-4633-5p, miR-6775-3p, miR-4749-3p, miR-323b-3p, miR-491-5p, and let-7 genes and probes on a Stratagene Mx3005P instrument (Agilent Technology, Santa Clara, CA, USA). U6 was used for miR normalization. The results were analyzed using the ΔΔ cycles to threshold (ΔΔCt). 

### 2.9. Statistical Analysis

Student’s t-test and one-way ANOVA test with Bonferroni correction were used to assess continuous data. Logistic regression analysis was performed using R software, version 4.0.5. Univariate analysis was performed to determine if patient age, sex, Filipino American or European American ethnicity, pTNM, and/or BMI were significant predictors of upregulation of miR-4633-5p. Once the statistical significance of the predictors was determined, multivariate logistic regression analysis was performed, and the interaction model was tested for significance. *P* values of less than 0.05 were considered to indicate statistical significance.

For the significant variables, odds ratios and their 95% confidence interval (CI) were calculated using the package “epitools”, version 0.5.10.1. For miR-4633-5p, we calculated the odds ratios, 95% CI, and *p* values. For the calculation of the odds ratio, we used the unconditional maximum likelihood estimate (MLE), and for the CI we used the normal approximation (Wald). The *p* values for the odds ratios were based on the chi-square statistics and declared significant for *p* values less than 0.05.

## 3. Results

### 3.1. Identifying the Differentially Expressed miRNA Signature in Filipino Americans Compared to European Americans

**Figure 2:**
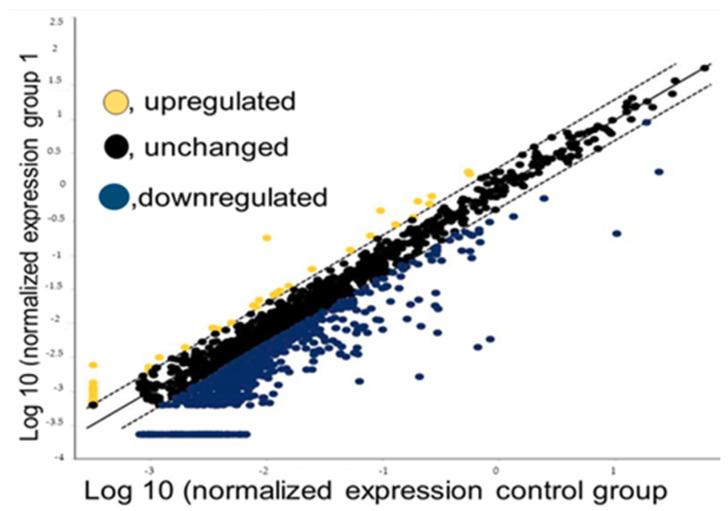
A scatter plot of miRNA-seq data normalized expression. Filipino American samples were used as tests and European American samples as controls. Data points beyond the dotted lines are shown in the upper left, and the lower right section meets the selected fold regulation threshold.

Our sequencing data showed that the vast majority of miRNAs are downregulated and unchanged, whereas only a few are upregulated, in Filipino Americans when compared to European Americans as positive controls (Figure 2). Using the scatter plot, we compared two groups: one as the control (five self-identified European American samples) and the other as a test group (five self-identified Filipino American samples). We analyzed our data to determine the differential expression of miRNAs using the data analysis web portal. The scatter plot (Figure 1) generated by the web portal compares the normalized expression of every gene on the array between the two selected groups by plotting them against one another to quickly visualize large changes in gene expression.

In our study, we observed that the number of miRNAs that were significantly downregulated in Filipino Americans when compared to European Americans was approximately nine times higher than that of the miRNAs that were significantly upregulated. There were 51 upregulated and 448 downregulated miRNAs in the Filipino American samples compared to the European American samples and the rest were unchanged in Filipino Americans when compared to European Americans. We ranked these miRNAs in the order of highest to lowest differential expression, and the top ten miRNAs that were down- or upregulated in the Filipino American patients when compared to the European American patients ranged from −97.34 to −444.88 folds (Table 1) and 2.74 to 40.06 folds (Table 2), respectively. The entire set of 51 miRNAs that were upregulated is listed in Supplemental Table S1 and the 448 miRNAs that were downregulated are listed in Supplemental Table S2. This approach allowed for simultaneous analysis of the expression patterns of a vast number of targeting genes.

### 3.2. Construction of miRNA–mRNA Interaction Network and Pathway Analysis Using Ingenuity Pathway Analysis (IPA)

IPA (QIAGEN), mapped 50 of the 51 miRNAs that were upregulated. The one that was missing in the IPA knowledgebase was hsa-miR-3653-3p. Similarly, the downregulated dataset contained 448 IDs, out of which 444 were mapped; hsa-miR-1254, hsa-miR-1273a, hsa-miR-3669, and hsa-miR-548-3p did not map with those in the IPA knowledgebase. Most of the differentially expressed miRNAs were linked to organismal injury and abnormalities, reproductive system disease, and cancer. The molecular and cellular functions associated with these miRNAs were cellular growth and proliferation, cell cycle, and cellular development (Table 3 and Table 4).

### 3.3. Analysis of miRNA Targets Using IPA

The lists of up- and downregulated miRNAs were used as input for IPA for the next phase of the analysis. We were able to detect the putative targets for almost all of the miRNAs that were differentially regulated in Filipino Americans compared to European Americans, using IPA target filter analysis that uses TargetScan as an inbuilt web-based miRNA target prediction tool. 

As mentioned before, 50 upregulated miRNAs out of 51 were mapped to the IPA knowledgebase. Using the “microRNA target filter analysis” option, 48 miRNAs were found to target 14,090 mRNAs. Next, we selected the thyroid cancer signaling pathway and found that 41 out of the 48 miRNAs were targeting 44 mRNAs within the thyroid cancer signaling pathway (Supplemental Table S3). Similarly, for the set of downregulated miRNAs, initially, 414 miRNAs were found to target 18368 mRNAs. After selecting thyroid cancer signaling, 341 out of the 414 miRNAs were found to target 51 mRNAs (Supplemental Table S4). The results show that hsa-miR-4731-3p, hsa-miR-3150a-3p, hsa-miR-4664-5p, and hsa-miR-6804-5p from the upregulated set were targeting the maximum number of mRNAs, 10, 8, 7, and 7, respectively, whereas from the downregulated set, hsa-miR-4731-5p was targeting 12 molecules, hsa-miR-491-5p was targeting 10 molecules, and hsa-let-7e-5p, hsa-miR-506-3p, hsa-miR-3689b-3p, and hsa-miR-5582-5p were each targeting 9 molecules. By IPA analysis, we identified upregulated and downregulated miRNA target genes related to diseases and biofunction, as shown in Table 3 and Table 4, respectively.

### 3.4. Analyses of Molecular Function and Enriched Canonical Pathways for miRNA Targets

Next, we added the thyroid cancer signaling pathway molecules in the “my pathways” tool for visualizing the direct and indirect relationships among the miRNAs and the molecules from the pathway. We used the “grow” tool and selected the confidence level to be either experimentally observed or predicted with high confidence. We found seven miRNAs from the upregulated set connected as the upstream or downstream of ten molecules from the thyroid cancer signaling pathway in humans. 

**Figure 3:**
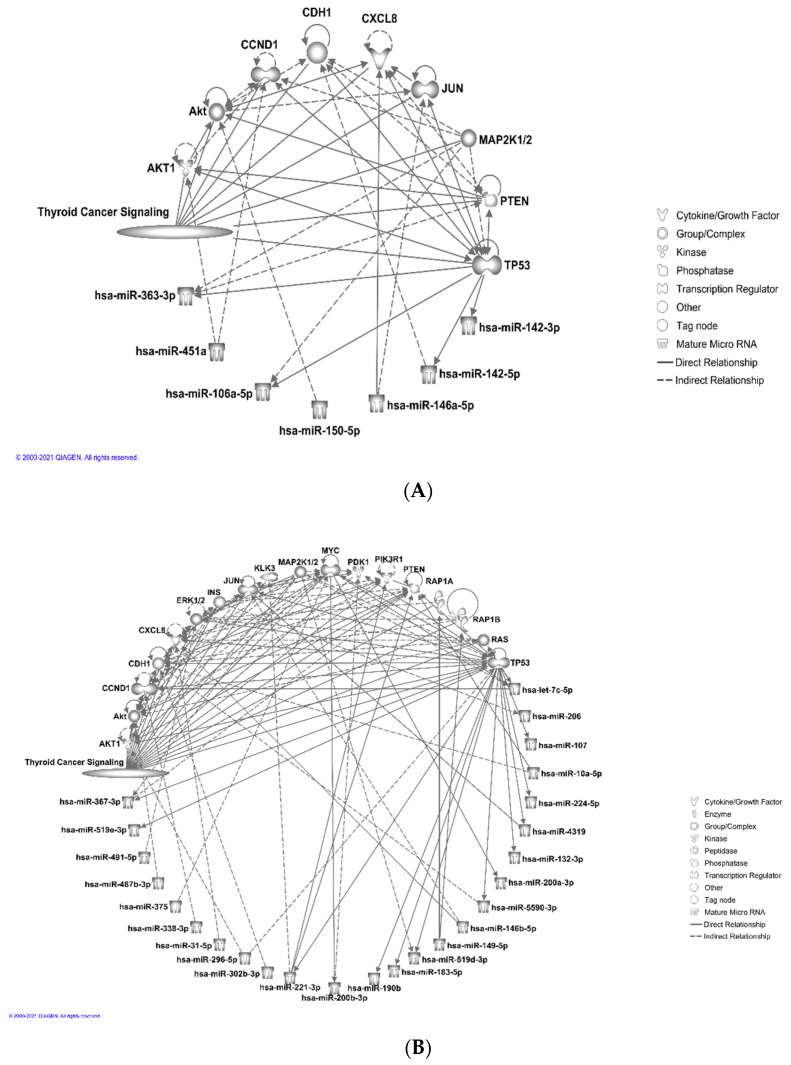
Ingenuity pathway analysis, IPA (© 2021-2021 QIAGEN) for visualization of direct and indirect relationship between miRNAs and their target genes within the thyroid signaling pathway. (**A**) Seven upregulated miRNAs are connected directly or indirectly to ten different molecules within the thyroid cancer signaling pathway. (**B**) Twenty five downregulated miRNAs are connected directly or indirectly to eighteen molecules within the thyroid cancer signaling pathway.

Then, we used the “connect” tool to incorporate more direct and indirect relationships among those molecules (Figure 3A). Similarly, for the set of downregulated miRNAs, using the thyroid cancer signaling pathway we found 25 miRNAs connected directly or indirectly with 18 molecules from the pathway (Figure 3B). The results suggested that in the case of humans, multiple downregulated miRNAs were targeting TP53, CCND1, and MYC genes, which act as hub genes within the thyroid cancer signaling pathway (Figure 3B). TP53 and CCND1 were also found to be targeted by multiple upregulated miRNAs (Figure 3A). In the case of all species, as expected, the number of interactions between the miRNA and the mRNAs increased for both the up- and downregulated sets (data not shown). We detected seven downregulated miRNAs that are linked significantly (*p* < 0.01) to PTC (Table 5).

**Figure 4:**
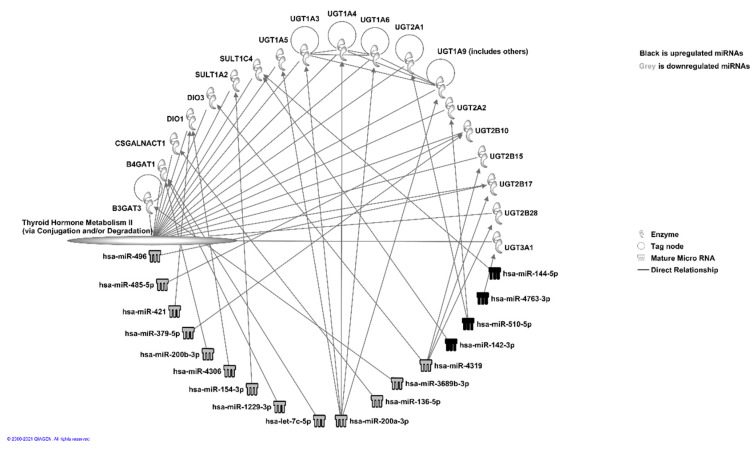
miRNA interaction analysis using IPA (© 2021-2021 QIAGEN) of miRNA Target Filtering program that uses results from TargetScan to identify the high-confidence putative miRNA targets. Figure shows miRNAs and their targets involved in thyroid hormone metabolic pathway II.

One of the miRNAs from downregulated sets was found to be linked significantly to the senescence of thyroid tumor cell lines (*p* < 0.05), and two were found to be linked to the proliferation of thyroid tumor cell lines (*p* < 0.05). We also observed that a number of miRNAs were linked to thyroid hormone metabolism pathways (Figure 4). 

**Figure 5:**
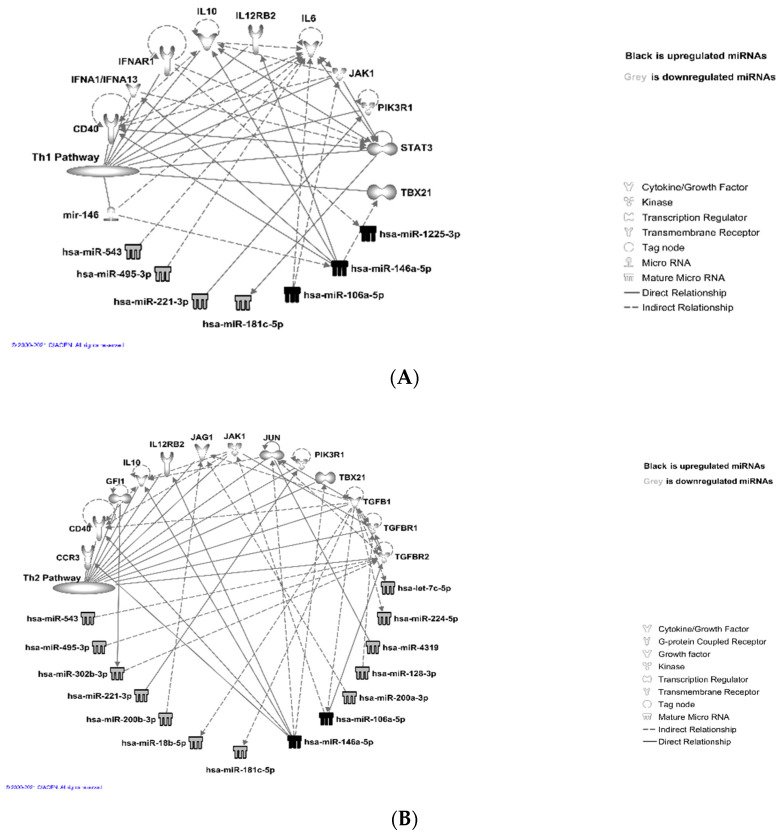

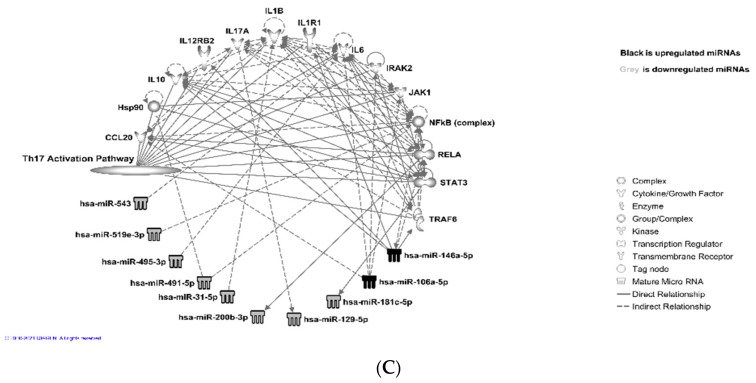
miRNAs targeting T-cell receptor signaling pathways: IPA (© 2021-2021 QIAGEN) analysis showed that these miRNAs target T-cell receptor signaling (both Th1 and Th2) and Th17 activation pathways: (**A**) T-helper 1 (Th1); (**B**) T-helper 2 (Th2); (**C**) T-helper 17 (Th17) activation pathway.

Further analysis showed that the upregulated miRNAs: miR-146a-5p, miR-1225-3p, miR106a-5p, and downregulated miRNAs: miR-543, miR-495-3p, miR-221-3p, target T-cell receptor signaling (Th1 pathway) via IL-10, IL-6, IL12RB2, JAK1/STAT3, PIK3R1, CD40, IFNAR1, and IFNA1/IFNA13, as shown in Figure 5A and Supplemental Table S5. Downregulated miRNAs: miR543, miR-495-3p, miR-221-3p, miR-200b-5p, miR-181c-5p, miR-200a-3p, miR-128-3p, miR-4319, miR-224-5p, and let-7a-5p, and upregulated miRNAs: miR-146a-5p and miR-106a 5p, are known to target Th2 via IL-10, IL-12, JUN, JAK1, JAG1, PIK3R1, TBX21, TGF b1, TGFBR1, CCR3, and CD40 genes, as shown in Figure 5B and Supplemental Table S6. The differentially expressed miRNAs miR-543, miR-519e-3p, miR-495-3p, miR-491-5p, miR-31-5p, miR-129-5p, miR-181c-5p, miR106a-5p, and miR-146a-5p were also found to be linked to the T-helper 17 (Th17) activation pathway (Figure 5C and Supplemental Table S7) through molecules such as JAK1/STATA3, RELA, CCL20, NFkB, and HSP90 complexes and a group of interleukins.

### 3.5. Analysis of Upregulated miRNA Signatures in Filipino Americans Compared to European Americans

**Figure 6:**
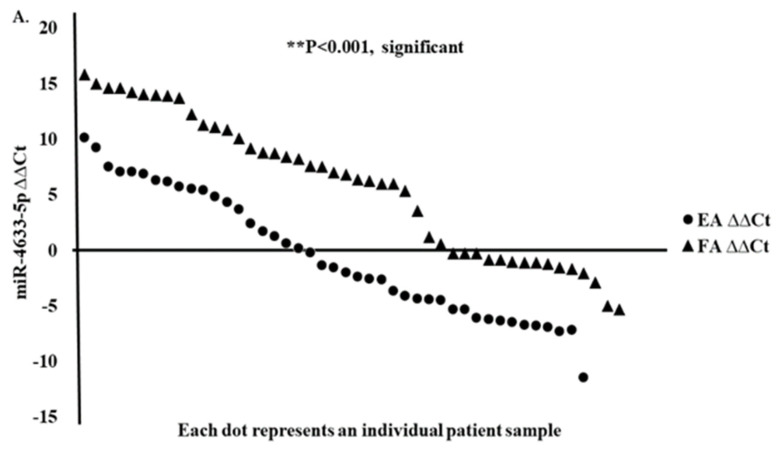

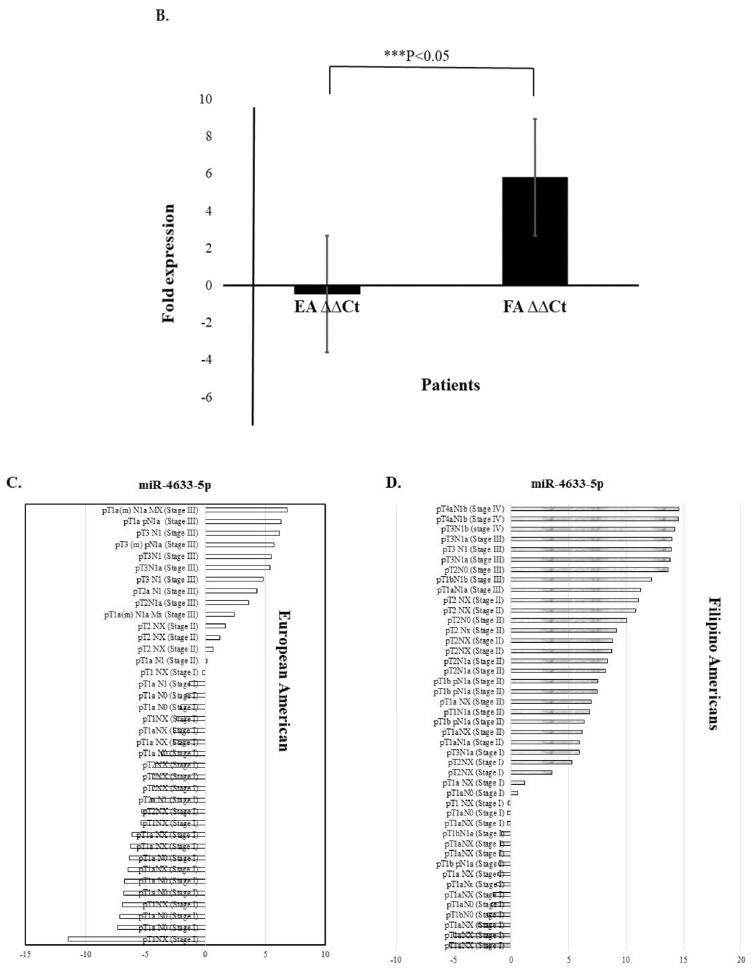
Upregulated miRNA signature validation by RT-qPCR. RT-qPCR assay for miRNA was performed with specific primers for miR-4633-5p gene. (**A**) Relative expression (ΔΔCt) and (**B**) fold expression values were compared in European Americans versus Filipino Americans. (**A**) Significantly (*** *p* < 0.0001) higher expression of miR-4633-5p was shown in Filipino Americans (*n* = 46) versus European Americans (*n* = 43). An increasing trend of higher expression of miR-4633-5p was observed in larger tumor size and staging (pTNM) in (**D**) Filipino Americans compared to (**C**) European Americans.

We confirmed by quantitative real-time PCR (RT-qPCR) the expressions of the top three up and downregulated miRNAs (upregulated miR-4633-5p, miR-6775-3p, and miR-4749-3p genes, and downregulated miR-323b-3p, miR-491-5p, and let-7 gene family) from RNA-seq data in Filipino American (*n* = 50) compared to European American (*n* = 50) samples and correlated the expression of selected miRNAs to disease stages. Interestingly, miR-4633-5p was consistently upregulated in Filipino American versus European American samples (Figure 6A,B), and was specifically correlated to the advanced stages (Table 6). Some of the samples from both ethnic groups showed downregulation. We found a significant correlation of miR-4633-5p upregulation (Figure 6A,B) with stages III and IV in Filipino Americans compared to European Americans (Figure 6C,D). 

A stage-dependent significantly higher miR-4633-5p expression (* *p* < 0.05) was shown to correlate in Filipino Americans with thyroid cancer (Figure 6D and Table 6).

In the univariate analysis, ethnicity (*p* = 0.0145), pTNM stage II (*p* = 0.0162), and pTNM stage III (*p* = 0.0298) were significant factors predicting miR-4633-5p, miR-6775-3p, and miR-4749-3p upregulation. When including these factors in a multivariate logistic regression model, ethnicity (*p* = 0.0262), pTNM II (*p* < 0.0441), and pTNM III (*p* < 0.0125) remained significant independent predictors of miRNA upregulation, whereas the interaction of staging and ethnicity was not significant. Table 6 shows the upregulation of miR-4633-5p among patients of different ethnicities and with different pTNM stages. The estimated odds ratio among Filipino Americans compared with European Americans was 3.03 (95% CI, 1.25-7.35), *p* = 0.013. For patients with pTNM stage I compared to pTNM stage II–IV, the odds ratio was 3.97 (95% CI, 1.29–12.22), *p* = 0.011. 

Upregulated miR-4633-5p target genes are shown in Supplemental Table S8. Patient demographics with raw data of miR-4633-5p are also shown in Supplemental Tables S9–S11. 

### 3.6. Analysis of Downregulated miRNA Signature in Filipino Americans Compared to European Americans

**Figure 7:**
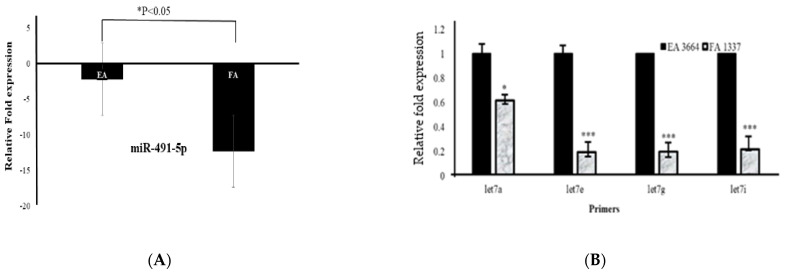
Downregulated miRNA signature validation by RT-qPCR. The RT-qPCR assays for miRNAs were performed with specific primers for miR-491-5p and let-7 genes. Relative downregulation (∆∆Ct) values of miR-491-5p and let-7 gene family are compared in European Americans (*n* = 43) versus Filipino Americans (*n* = 46). Significantly lower expression is noted in Filipino Americans compared to European Americans. (**A**) miR-491-5p (*** *p* < 0.001) and (**B**) let-7 subfamilies: let-7a (* *p* < 0.05), let-7e (*** *p* < 0.001), let-7g (*** *p* < 0.001), let-7i (*** *p* < 0.001).

We confirmed that miR-491-5p and let-7 gene families were downregulated (Figure 7A,B) in Filipino Americans compared to European Americans. We performed RT-qPCR of the same miRNA samples that were used for Hiseq4000. In contrast, ethnicity remained an independent predictor of significantly downregulated miR-491-5p and let-7 family genes irrespective of tumor staging. We found that miR-491-5p (Figure 7A) and the let-7 family, specifically let-7a, let-7e, let-7g, and let-7i (Figure 7B), were significantly downregulated (* *p* < 0.05) in the Filipino American compared to European American samples in all stages of cancer. Target genes for miR-491-5p and let-7 genes are shown in Supplemental Tables S12 and S13.

Although miR-6775-3p and miR-4749-3p were variably upregulated and showed no difference in ethnicity, staging and upregulation of miR-6775-3p and miR-4749-3p showed an independent predicting factor (Supplemental Figures S1 and S2). Although miR-323b-3p showed downregulation in Filipino American samples compared to European American samples, the reverse was observed in RT-qPCR; most of them showed upregulation in both ethnic groups (Supplemental Figure S3) and most of the samples showed downregulation of miR-491-5p (Supplemental Figure S4).

## 4. Discussion

In this study, we applied small RNA-seq to examine tissue-specific miRNA profiles from Filipino American and European American patients with thyroid cancer to understand the differential expression patterns and to evaluate their abundance, targets, and relation to different biological pathways. We were able to prepare a clean miRNA library from the archival samples. 

Using IPA, we have shown that relevant pathways are likely regulated by the targets of highly conserved miRNAs, which are differentially expressed in Filipino and European Americans. We were able to detect the putative targets for most of these miRNAs by IPA. We have analyzed the miRNA target genes that are related to human diseases and are involved functionally in disease progression. The targeted pathways involve kinases and transmembrane receptor molecules (downregulated let-7e, miR-125, miR-134, miR-140, miR-143) (upregulated miR-1, miR-122, miR-130, miR-132, miR-133b, miR-142-5p), which are involved in the cell cycle and cellular differentiation/cell proliferation. We have shown a large number of downregulated genes that are involved in organismal injury and abnormalities, cancer, gastrointestinal disease, cellular development, and immunological disorders. The upregulated clusters were fewer in number and are known to be involved in immunological disorders, neurological development (cell cycle), and urological disorders. We detected seven of the known downregulated miRNAs (miR-125b-2-3p, miR-130a-3p, miR-17-5p, miR181a-5p, miR-221-3p, miR-31-5p, miR-381-3p) linked to papillary thyroid cancer or cell lines (miR-17-5p) with tumor suppressor or oncogenic function reported in the literature. IPA canonical pathway analysis was used for the prediction of pathways that are activated or inhibited. Pathway maps were used to understand the correlation between miRNA target genes and different diseases so that this can lead to new targets for therapeutic agents in the future. Additionally, we have detected that the targets for the differentially expressed miRNAs are involved in pathways related to thyroid hormone metabolism. Although thyroid hormones regulate the physiological processes of normal cells, thyroid hormones and their receptors are dysregulated in thyroid cancer. Therefore, it will be interesting to see whether the influence of miRNAs targeting the metabolic pathways affect thyroid cancer health disparities. 

It has been shown that inflammation and immunity alter the prognosis in many cancers, including thyroid cancer. Recent data showed that the ratio of immune cells plays an important role in the prognosis of high-risk PTC. Interestingly, our data showed that the differentially regulated miRNAs with their targets are linked to T cell receptor signaling pathways (T-helper type 1 and 2; Th1 and Th2 signaling pathways). Further study is needed to elucidate the roles of Th1 and Th2 pathways in thyroid cancer progression in Filipino Americans with aggressive PTC. We also found that the targets of two upregulated and eight downregulated miRNAs were linked to the T-helper type 17 (Th17) activation pathway. Th17 cells are known to influence anti-tumoral CD8+ T cell responses. Although Th17 cells play controversial roles in cancer due to their dual roles, either good or bad in cancer progression, it is important to determine whether they are cancer type-specific or influenced by the tumor microenvironment. Further studies are needed to reveal the significance of these pathways.

Out of all miRNAs, we chose the top three upregulated ones—miR-4633-5p [24,25,26], miR-6775-3p [27], and miR-4749-3p [28]. Although miR-323b-3p was downregulated in sequencing data, the reverse was observed when we performed RT-qPCR; the majority of samples showed miR-323b-3p upregulation with very few downregulated ones. These discrepancies need to be investigated in more samples. The other two most relevant downregulated miRNAs were miR-491-5p [19,29,30,31,32,33,34,35,36,37,38,39,40] and the let-7 gene family [37,39,40,41,42]. Several target genes were detected for miR-4633-5p, miR-323-3p, and miR-491-5p or let-7 family genes in cancer and other diseases; however, very few studies have shown their involvement in thyroid cancer. In melanoma, miR-4633-5p has been shown to act as a tumor suppressor gene, suppressing the activation of the Akt pathway and the secretion of MMP2 [25]; in contrast, consistently higher miR-4633-5p showed an association with advanced thyroid cancer staging. Target genes for miR-4633-5p/miR-323-3p showed interaction with the PDZ and LIM domain families, which we recently published as an important oncogene, called Enigma [43]. On the other hand, although miR-323b-3p was found to be downregulated in Filipino Americans versus European Americans by miRNA-seq data, interestingly, only a few samples showed downregulation, and the majority of the samples showed an upregulation in Filipino Americans and no significant difference from the European American miRNA samples. We hypothesize that because fewer samples were used for miRNA sequencing, discrepancies were observed in the RT-qPCR data from a larger cohort of patient samples. miR-323-3p is dysregulated in a variety of human cancers, including thyroid [44], lung [42], pancreatic [45], and prostate cancer. Although we found no difference in expression between the two ethnicities, miR-323b-3p upregulation indicated an oncogenic miRNA in thyroid cancer. miR-323-3p targets one of the PDLIM family genes, out of which we have observed that PDLIM7 (Enigma) is upregulated in the same thyroid cancer tissues but not in benign tumors via the activation of MAPK and c-MYC (unpublished data). 

Previous studies showed lower expressions of miRNA-491-5p and let-7 in individual tumors [19,31,33,34,39,40,41,46,47]. The exact regulatory mechanisms of these miRNAs are still not clear [48,49]. One of the most interesting pathways of miR-491-5p as a tumor suppressor is by targeting the Wnt3/β-catenin pathway mediated via Foxi1 [33]. On the other hand, let-7 family genes are involved in thyroid cancer oncogenesis [41]. Multiple studies have shown that let-7 family genes are used as prognostic markers, and they play important roles in thyroid cancer cell proliferation, invasion, and metastasis [37,39,40,41,47]. The proposed mechanisms of let-7 in thyroid and other cancers are shown to be via targeting AKT, SNAIL upregulation or the suppression of immune evasion [41,48]. Another study showed that the downregulation of miR-491-5p also promotes the metastasis of gastric cancer via SNAIL and FGF4 [46]. In particular, two miRNAs (miR-491-5p and let7) were consistently downregulated in these tumor tissues from Filipino Americans versus European Americans. Both miR-491-5p and let-7 can target SNAIL, acting as tumor suppressor miRNAs. Therefore, we are interested to see whether there are any functional implications of these miRNAs in thyroid cancer health disparities. 

In conclusion, we have detected both up- and downregulated miRNA signatures in Filipino Americans when compared to European Americans by Hiseq4000 and confirmed the differential expressions by RT-qPCR in archival tissue samples. We have also identified target genes for these miRNAs and the pathways to which those are related by IPA. Out of the top ten up- and downregulated miRNAs, we found only a few of them are significantly differentially expressed with advanced thyroid cancer staging. Future study is underway to validate the functional implications of the upregulated miR-4633-5p/miR-323-3p genes and downregulated miR-4915p/let-7 family genes and their involvement in the pathways related to thyroid cancer progression, not only in Filipino Americans but also in other ethnic groups.

## Figures and Tables

**Table 1:** Top ten downregulated miRNAs (FA vs. EA).

Name of the miRNA	Differential Expression in FA vs. EA
hsa-miR-323b-3p	−444.88
hsa-miR-495-5p	−160.46
hsa-miR-412-5p	−158.80
hsa-miR-431-5p	−136.01
hsa-miR-375	−129.45
hsa-miR-4471	−120.93
hsa-miR-129-2-3p	−112.39
hsa-miR-4299	−111.30
hsa-miR-129-5p	−101.76
hsa-miR-4662a-5p	−97.34

FA, Filipino Americans; EA, European Americans.

**Table 2:** Top ten upregulated miRNAs (FA vs. EA).

Name of the miRNA	Differential Expression in FA vs. EA
hsa-miR-4633-5p	40.06
hsa-miR-4749-3p	38.20
hsa-miR-526b-5p	34.11
hsa-miR-142-3p	11.14
hsa-miR-142-5p	9.27
hsa-miR-150-5p	9.00
hsa-miR-146a-5p	4.31
hsa-miR-6775-3p	3.10
hsa-miR-548as-5p	2.79
hsa-miR-105-3p	2.74

FA, Filipino Americans; EA, European Americans.

**Table 3:** Top diseases and bio functions related to upregulated miRNA target genes.

	Top Diseases and Bio Functions	*p*-Value Range	No. of Molecules
Diseases and disorders	Organismal injury and abnormalities	4.90 × 10^−2^–1.30 × 10^−11^	19
	Reproductive system disease	4.90 × 10^−2^–1.30 × 10^−11^	15
	Cancer	4.90 × 10^−2^–2.12 × 10^−9^	15
	Hematological disease	3.36 × 10^−2^–2.12 × 10^−9^	9
	Immunological disease	3.36 × 10^−2^–2.12 × 10^−9^	10
Molecular and cellular functions	Cellular growth and proliferation	4.82 × 10^−2^–1.19 × 10^−5^	8
	Cell cycle	3.53 × 10^−2^–1.51 × 10^−4^	5
	Cellular development	4.98 × 10^−2^–9.58 × 10^−4^	8
	DNA replication, recombination, and repair	2.58 × 10^−2^–1.67 × 10^−3^	3
	Cell morphology	1.66 × 10^−2^–2.50 × 10^−3^	2

**Table 4:** Top diseases and bio functions related to downregulated miRNA target genes.

	Top Diseases and Bio Functions	*p*-Value Range	No. of Molecules
Diseases and disorders	Organismal injury and abnormalities	4.75 × 10^−2^–2.25 × 10^−61^	121
	Reproductive system disease	3.59 × 10^−2^–2.25 × 10^−61^	82
	Cancer	4.75 × 10^−2^–6.25 × 10^−20^	67
	Inflammatory disease	3.23 × 10^−2^–6.22 × 10^−19^	40
	Inflammatory response	1.81 × 10^−2^–6.22 × 10^−19^	35
Molecular and cellular functions	Cell cycle	3.00 × 10^−2^–1.19 × 10^−8^	11
	Cellular movement	4.63 × 10^−2^–1.19 × 10^−8^	29
	Cellular growth and proliferation	5.00 × 10^−2^–3.25 × 10^−4^	38
	Cellular development	5.00 × 10^−2^–6.59 × 10^−4^	38
	Cell death and survival	4.75 × 10^−2^–7.80 × 10^−3^	6

**Table 5:** Downregulated miRNAs linked to thyroid cancer.

Categories	Diseases or Function Annotation	*p*-Value	Molecules	No. of Molecules
Cancer, endocrine system disorders, organismal injury, and abnormalities	Papillary thyroid carcinoma	0.000294	miR-125b-2-3p (and other miRNAs w/seed CAAGUCA), miR-130a-3p (and other miRNAs w/seed AGUGCAA), miR-17-5p (and other miRNAs w/seed AAAGUGC), miR-181a-5p (and other miRNAs w/seed ACAUUCA), miR-221-3p (and other miRNAs w/seed GCUACAU), miR-31-5p (and other miRNAs w/seed GGCAAGA), miR-381-3p (and other miRNAs w/seed AUACAAG)	7
Cell cycle	Senescence of thyroid tumor cell lines	0.0241	miR-17-5p (and other miRNAs w/seed AAAGUGC)	1
Cellular development, cellular growth, and proliferation	Proliferation of thyroid tumor cell lines	0.05	miR-17-5p (and other miRNAs w/seed AAAGUGC), miR-18a-5p (and other miRNAs w/seed AAGGUGC)	2

**Table 6:** Analysis of the upregulated miR-4633-5p among patients from different ethnicities and different pTNM staging.

Odds Ratios for Upregulated miR-4633-5p		
Factor	Odds Ratio (95% CI)	*p*-Value
Ethnicity: FA versus EA	3.03 (1.25–7.35)	0.013 *
pTNM staging: I versus II, III, and IV	3.97 (1.29–12.22)	0.011 *

* *p* < 0.05, statistically significant; CI, confidence interval; FA, Filipino American; EA, European American; pTNM, pathological tumor node metastasis.

## Data Availability

Some datasets generated during and/or analyzed during the current study are not publicly available but are available from the corresponding author on reasonable request.

## Supplementary Materials

The following are available online at https://www.mdpi.com/article/10.3390/curroncol28050309/s1. Figure S1: miR-6775-3p was upregulated with no significant difference observed between the patients from the two different ethnicities (*p* = 0.431, not significant)*,* Figure S2: miR-4749-3p was upregulated with no significant difference observed between the patients from the two different ethnicities (*p* = 0.485, not significant), Figure S3: miR-323b-3p was upregulated instead of downregulated in most of the samples with no significant difference observed between the patients from the two different ethnicities (*p* = 0.299, not significant), Figure S4: miR-491-5p was downregulated significantly in Filipino Americans compared to the European Americans (*** *p* < 0.001, statistically significant), Table S1: List of 51 upregulated miRNAs sorted by fold change, Table S2: List of 451 downregulated miRNAs sorted by fold change, Table S3: List of 44 target mRNAs for 41 distinctive upregulated miRNAs (includes all confidence levels), Table S4: List of 51 target mRNAs for 341 distinctive downregulated miRNAs (includes all confidence levels), Table S5: Relationships between the molecules (miRNAs and mRNAs) within the Th1 pathway, Table S6: Relationships between the molecules (miRNAs and mRNAs) within the Th2 pathway, Table S7: Relationships between the molecules (miRNAs and mRNAs) within the Th17 pathway, Table S8: Upregulated miR-4633-5p-target genes in cancers, Table S9: Patient demographic of TNM (T1) staging with age, sex, BMI with miR-4633-5p expression in Filipinos vs. Europeans, Table S10: Patient demographic of TNM (T2) staging with age, sex, BMI with miR-4633-5p expression in Filipinos vs. Europeans, Table S11: Correlation of clinicopathological features with TNM (T3) staging age, sex, with miR-4633-5p expression in Filipinos vs. European, Table S12: Downregulated miR-491-5p-target genes in cancers, Table S13: Upregulated let-7 family-target genes in cancers.
