# Supplementary material for: Differential Expression of Non-Coding RNA Signatures in Thyroid Cancer between Two Ethnic Groups
Source: Curr Oncol. 2021 Sep 19;28(5):3610–28. doi: 10.3390/curroncol28050309 (PMC8482137; doi:10.3390/curroncol28050309)
Supplement: Supplementary file 1 [file curroncol-28-00309-s001.zip › curroncol-1329583-supplementary.pdf]

Supplemental Materials:

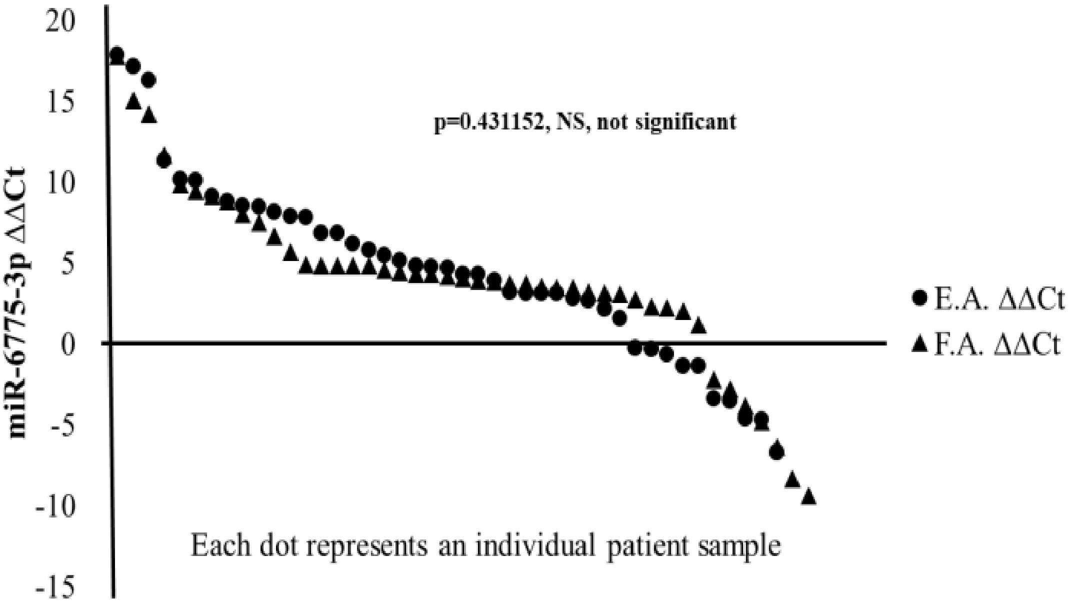

Figure S1. miR-6775-3p was upregulated with no significant difference observed between the patients from the two different ethnicities (p=0.431, not significant).

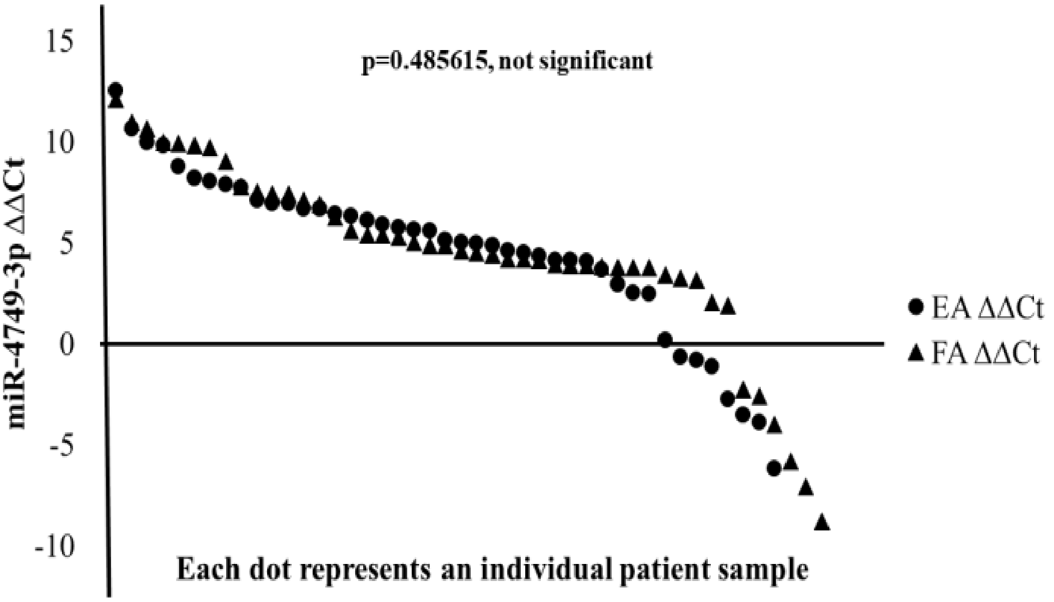

Figure S2. miR-4749-3p was upregulated with no significant difference observed between the patients from the two different ethnicities (p=0.485, not significant).

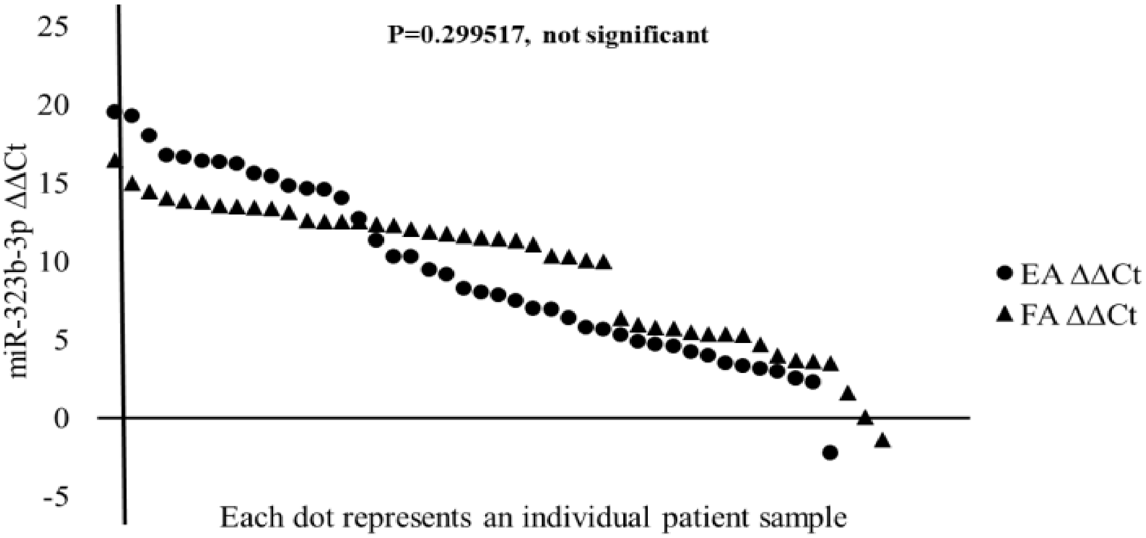

Figure S3. miR-323b-3p was upregulated instead of downregulated in most of the samples with no significant difference observed between the patients from the two different ethnicities (p=0.299, not significant).

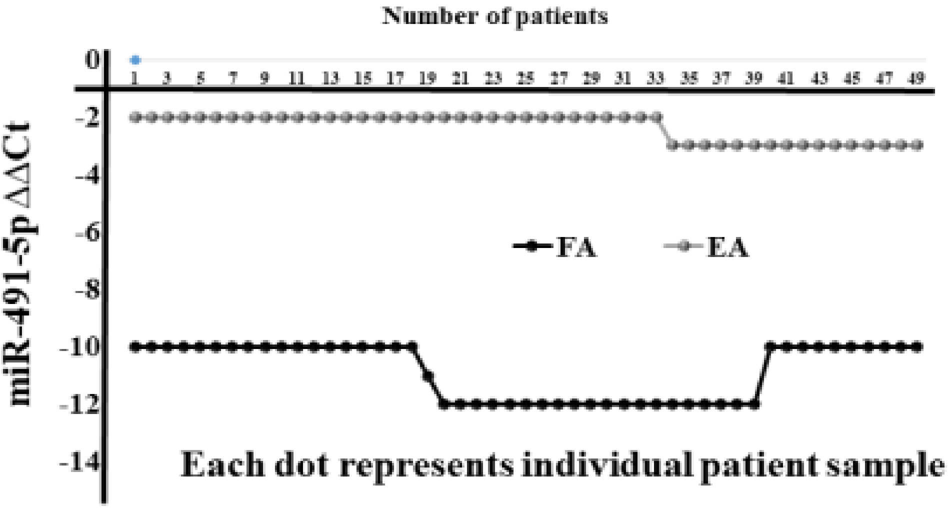

Figure S4. miR-491-5p was downregulated significantly in Filipino Americans compared to the European Americans (***p<0.001, statistically significant).

Table S1: List of 51 upregulated miRNAs sorted by fold change

#	miRNAs	Fold Change
1	hsa-miR-4633-5p	40.06
2	hsa-miR-4749-3p	38.20
3	hsa-miR-526b-5p	34.11
4	hsa-miR-142-3p	11.14
5	hsa-miR-142-5p	9.27
6	hsa-miR-150-5p	9.00
7	hsa-miR-146a-5p	4.31
8	hsa-miR-6775-3p	3.10
9	hsa-miR-548as-5p	2.79
10	hsa-miR-105-3p	2.74
11	hsa-miR-6848-5p	2.69
12	hsa-miR-518d-3p	2.66
13	hsa-miR-4999-5p	2.56
14	hsa-miR-510-5p	2.51
15	hsa-miR-451a	2.46
16	hsa-miR-7112-3p	2.46
17	hsa-miR-3150a-3p	2.43
18	hsa-miR-363-3p	2.40
19	hsa-miR-4454	2.37
20	hsa-miR-1225-3p	2.36
21	hsa-miR-6758-5p	2.36
22	hsa-miR-767-5p	2.36
23	hsa-miR-548a	2.31
24	hsa-miR-6813-3p	2.29
25	hsa-miR-144-5p	2.27
26	hsa-miR-6804-5p	2.26
27	hsa-miR-455-5p	2.25
28	hsa-miR-3185	2.24
29	hsa-miR-3975	2.23
30	hsa-miR-4653-5p	2.22
31	hsa-miR-6805-5p	2.22
32	hsa-miR-548ae-5p	2.21
33	hsa-miR-6848-3p	2.21

34	hsa-miR-4763-3p	2.18
35	hsa-miR-6794-5p	2.18
36	hsa-miR-4310	2.17
37	hsa-miR-9500	2.14
38	hsa-miR-6081	2.13
39	hsa-miR-708-5p	2.12
40	hsa-miR-3653-3p	2.11
41	hsa-miR-548az-3p	2.11
42	hsa-miR-4722-3p	2.10
43	hsa-miR-645	2.08
44	hsa-miR-1539	2.07
45	hsa-miR-1292-5p	2.06
46	hsa-miR-3616-3p	2.06
47	hsa-miR-4524b-3p	2.05
48	hsa-miR-548ae-3p	2.05
49	hsa-miR-5583-3p	2.05
50	hsa-miR-106a-5p	2.02
51	hsa-miR-4664-5p	2.01

Table S2: List of 451 downregulated miRNAs sorted by fold change

#	miRNAs	Fold Change
1	hsa-miR-323b-3p	-444.88
2	hsa-miR-495-5p	-160.46
3	hsa-miR-412-5p	-158.80
4	hsa-miR-431-5p	-136.01
5	hsa-miR-375	-129.45
6	hsa-miR-4471	-120.93
7	hsa-miR-129-2-3p	-112.39
8	hsa-miR-4299	-111.30
9	hsa-miR-129-5p	-101.76
10	hsa-miR-4662a-5p	-97.34
11	hsa-miR-1185-1-3p	-89.03
12	hsa-miR-4539	-85.96
13	hsa-miR-379-3p	-84.16
14	hsa-miR-4804-5p	-83.93

15	hsa-miR-4712-3p	-83.43
16	hsa-miR-124-5p	-83.11
17	hsa-miR-154-3p	-80.68
18	hsa-miR-323a-3p	-80.21
19	hsa-miR-381-5p	-80.15
20	hsa-miR-297	-72.21
21	hsa-miR-4315	-71.13
22	hsa-miR-3652	-70.97
23	hsa-miR-380-3p	-69.72
24	hsa-miR-1271-3p	-69.65
25	hsa-miR-4719	-68.97
26	hsa-miR-4694-3p	-68.26
27	hsa-miR-1197	-67.09
28	hsa-miR-4300	-66.50
29	hsa-miR-4319	-66.39
30	hsa-miR-4711-5p	-63.15
31	hsa-miR-4703-3p	-61.76
32	hsa-miR-376b-5p	-61.17
33	hsa-miR-6874-3p	-60.22
34	hsa-miR-4781-3p	-60.21
35	hsa-miR-3674	-60.18
36	hsa-miR-5687	-60.17
37	hsa-miR-302b-3p	-59.17
38	hsa-miR-4645-3p	-58.92
39	hsa-miR-4451	-58.45
40	hsa-miR-384	-58.21
41	hsa-miR-4303	-57.27
42	hsa-miR-4745-3p	-54.80
43	hsa-miR-647	-53.75
44	hsa-miR-4678	-53.63
45	hsa-miR-3672	-53.41
46	hsa-miR-448	-53.37
47	hsa-miR-4766-3p	-53.34
48	hsa-miR-1288-3p	-53.09
49	hsa-miR-2052	-52.68

50	hsa-miR-3973	-52.68
51	hsa-miR-3130-5p	-52.61
52	hsa-miR-4495	-52.59
53	hsa-miR-4803	-52.39
54	hsa-miR-1245b-5p	-52.29
55	hsa-miR-3650	-51.84
56	hsa-miR-5706	-50.39
57	hsa-miR-216b-3p	-50.02
58	hsa-miR-6500-3p	-50.02
59	hsa-miR-513c-3p	-49.69
60	hsa-miR-122-3p	-49.54
61	hsa-miR-548ag	-49.24
62	hsa-miR-6755-3p	-49.21
63	hsa-miR-2053	-47.35
64	hsa-miR-562	-46.59
65	hsa-miR-18b-5p	-45.74
66	hsa-miR-1288-5p	-45.67
67	hsa-miR-569	-45.39
68	hsa-miR-4724-3p	-45.25
69	hsa-miR-4264	-44.08
70	hsa-miR-3689e	-43.97
71	hsa-miR-4715-3p	-43.91
72	hsa-miR-676-5p	-43.89
73	hsa-miR-4282	-43.88
74	hsa-miR-4782-3p	-43.45
75	hsa-miR-372-5p	-43.01
76	hsa-miR-1207-3p	-42.87
77	hsa-miR-4648	-42.85
78	hsa-miR-5590-5p	-42.67
79	hsa-miR-6855-3p	-42.36
80	hsa-miR-1178-5p	-42.28
81	hsa-miR-7106-3p	-42.21
82	hsa-miR-3121-5p	-41.56
83	hsa-miR-495-3p	-41.47
84	hsa-miR-5094	-40.50

85	hsa-miR-4757-3p	-40.46
86	hsa-miR-3136-3p	-40.11
87	hsa-miR-4709-3p	-40.05
88	hsa-miR-4699-3p	-39.89
89	hsa-miR-3978	-39.80
90	hsa-miR-1258	-38.97
91	hsa-miR-6513-5p	-38.62
92	hsa-miR-216b-5p	-37.89
93	hsa-miR-1266-3p	-36.98
94	hsa-miR-580-3p	-36.88
95	hsa-miR-573	-35.93
96	hsa-miR-3119	-35.88
97	hsa-miR-4420	-35.83
98	hsa-miR-6878-3p	-35.09
99	hsa-miR-3619-5p	-34.08
100	hsa-miR-302a-5p	-33.60
101	hsa-miR-487b-3p	-33.58
102	hsa-miR-4424	-33.57
103	hsa-miR-5582-3p	-32.63
104	hsa-miR-1256	-32.56
105	hsa-miR-380-5p	-31.85
106	hsa-miR-1245b-3p	-31.83
107	hsa-miR-3678-5p	-31.58
108	hsa-miR-3165	-31.45
109	hsa-miR-6736-5p	-30.96
110	hsa-miR-6502-5p	-30.66
111	hsa-miR-6854-3p	-30.59
112	hsa-miR-3129-3p	-30.17
113	hsa-miR-153-3p	-30.00
114	hsa-miR-4697-3p	-29.66
115	hsa-miR-588	-29.39
116	hsa-miR-3157-3p	-29.07
117	hsa-miR-215-3p	-29.06
118	hsa-miR-3177-5p	-28.64
119	hsa-miR-6720-3p	-28.31

120	hsa-miR-4666a-3p	-28.30
121	hsa-miR-6499-3p	-27.62
122	hsa-miR-4439	-27.38
123	hsa-miR-4727-5p	-27.22
124	hsa-miR-208a-3p	-27.16
125	hsa-miR-208b-5p	-26.91
126	hsa-miR-24-1-5p	-26.90
127	hsa-miR-6508-3p	-26.88
128	hsa-miR-3160-3p	-26.50
129	hsa-miR-4799-3p	-26.42
130	hsa-miR-6511b-5p	-26.39
131	hsa-miR-224-3p	-26.34
132	hsa-miR-579-5p	-26.13
133	hsa-miR-4671-5p	-25.62
134	hsa-miR-520a-5p	-25.61
135	hsa-miR-3681-3p	-25.39
136	hsa-miR-3689b-3p	-24.91
137	hsa-miR-587	-24.81
138	hsa-miR-4323	-24.39
139	hsa-miR-3126-3p	-24.37
140	hsa-miR-1193	-24.17
141	hsa-miR-5590-3p	-23.96
142	hsa-miR-4526	-23.79
143	hsa-miR-556-3p	-23.67
144	hsa-miR-5681a	-23.50
145	hsa-miR-4735-5p	-23.18
146	hsa-miR-5584-3p	-23.13
147	hsa-miR-6129	-22.91
148	hsa-miR-3135a	-22.79
149	hsa-miR-4276	-22.76
150	hsa-miR-4735-3p	-22.70
151	hsa-miR-4283	-22.68
152	hsa-miR-4718	-22.08
153	hsa-miR-621	-22.01
154	hsa-miR-1263	-21.96

155	hsa-miR-433-5p	-21.82
156	hsa-miR-556-5p	-21.24
157	hsa-miR-651-3p	-21.05
158	hsa-miR-5692b	-20.94
159	hsa-miR-4672	-20.57
160	hsa-miR-367-3p	-20.54
161	hsa-miR-4278	-20.35
162	hsa-miR-3677-3p	-19.46
163	hsa-miR-550a-3-5p	-19.43
164	hsa-miR-5704	-19.41
165	hsa-miR-603	-19.34
166	hsa-miR-3591-5p	-19.16
167	hsa-miR-4637	-19.09
168	hsa-miR-3680-5p	-18.76
169	hsa-miR-548ah-5p	-18.76
170	hsa-miR-4696	-18.72
171	hsa-miR-4795-5p	-18.65
172	hsa-miR-4692	-18.53
173	hsa-miR-518c-3p	-18.43
174	hsa-miR-520d-5p	-17.69
175	hsa-miR-4432	-17.31
176	hsa-miR-6744-3p	-17.23
177	hsa-miR-4733-5p	-17.12
178	hsa-miR-7-5p	-17.09
179	hsa-miR-4318	-17.08
180	hsa-miR-3191-5p	-16.54
181	hsa-miR-525-5p	-16.51
182	hsa-miR-4272	-16.00
183	hsa-miR-4639-3p	-16.00
184	hsa-miR-1278	-15.93
185	hsa-miR-4446-3p	-15.80
186	hsa-miR-4477a	-15.72
187	hsa-miR-302e	-15.50
188	hsa-miR-371a-3p	-15.22
189	hsa-miR-6841-3p	-15.15

190	hsa-miR-5707	-14.85
191	hsa-miR-1184	-14.75
192	hsa-miR-4761-5p	-14.71
193	hsa-miR-5580-3p	-14.70
194	hsa-miR-382-5p	-14.63
195	hsa-miR-4445-5p	-14.49
196	hsa-miR-6761-5p	-14.39
197	hsa-miR-4731-5p	-14.19
198	hsa-miR-409-3p	-13.98
199	hsa-miR-4720-5p	-13.97
200	hsa-miR-4720-3p	-13.74
201	hsa-miR-6853-3p	-13.53
202	hsa-miR-129-1-3p	-13.48
203	hsa-miR-5582-5p	-13.48
204	hsa-miR-604	-13.38
205	hsa-miR-4789-5p	-13.36
206	hsa-miR-3688-3p	-13.19
207	hsa-miR-4256	-12.89
208	hsa-miR-550b-2-5p	-12.23
209	hsa-miR-5692c	-12.15
210	hsa-miR-506-3p	-11.97
211	hsa-miR-19b-2-5p	-11.73
212	hsa-miR-4306	-11.73
213	hsa-miR-4486	-11.73
214	hsa-miR-525-3p	-11.73
215	hsa-miR-6509-5p	-11.70
216	hsa-miR-1269b	-11.65
217	hsa-miR-494-3p	-11.61
218	hsa-miR-5583-5p	-11.52
219	hsa-miR-485-3p	-11.49
220	hsa-miR-489-5p	-11.28
221	hsa-miR-6757-3p	-11.28
222	hsa-miR-670-3p	-11.27
223	hsa-miR-5579-3p	-11.17
224	hsa-miR-518a-3p	-11.15

225	hsa-miR-4506	-11.07
226	hsa-miR-4316	-11.00
227	hsa-miR-6738-3p	-10.97
228	hsa-miR-5092	-10.85
229	hsa-miR-3669	-10.81
230	hsa-miR-4666a-5p	-10.81
231	hsa-miR-499b-5p	-10.81
232	hsa-miR-6501-3p	-10.64
233	hsa-miR-4798-3p	-10.43
234	hsa-miR-518f-3p	-10.43
235	hsa-miR-4266	-10.26
236	hsa-miR-1973	-10.20
237	hsa-miR-5591-3p	-10.20
238	hsa-miR-548-3p	-10.03
239	hsa-miR-548aj-3p	-10.03
240	hsa-miR-655-5p	-10.03
241	hsa-miR-369-5p	-9.62
242	hsa-miR-1229-3p	-9.61
243	hsa-miR-20b-3p	-9.61
244	hsa-miR-519b-3p	-9.61
245	hsa-miR-519d-3p	-9.61
246	hsa-miR-519e-3p	-9.61
247	hsa-miR-5591-5p	-9.61
248	hsa-miR-488-3p	-9.50
249	hsa-miR-134-5p	-9.24
250	hsa-miR-1245a	-9.16
251	hsa-miR-4520-2-3p	-9.12
252	hsa-miR-485-5p	-8.96
253	hsa-miR-10a-3p	-8.94
254	hsa-miR-190b	-8.63
255	hsa-miR-409-5p	-8.26
256	hsa-miR-4756-5p	-8.04
257	hsa-miR-381-3p	-8.02
258	hsa-miR-433-3p	-7.69
259	hsa-miR-379-5p	-7.64

260	hsa-miR-369-3p	-7.41
261	hsa-miR-1185-5p	-7.30
262	hsa-miR-376c-3p	-7.28
263	hsa-miR-1471	-7.17
264	hsa-miR-329-3p	-7.10
265	hsa-miR-10a-5p	-6.96
266	hsa-miR-337-5p	-6.75
267	hsa-miR-154-5p	-6.73
268	hsa-miR-654-3p	-6.72
269	hsa-miR-410-3p	-6.71
270	hsa-miR-370-3p	-6.51
271	hsa-miR-1199-3p	-6.44
272	hsa-miR-376a-3p	-6.43
273	hsa-miR-1254	-6.19
274	hsa-miR-335-5p	-6.16
275	hsa-miR-431-3p	-6.05
276	hsa-miR-1180-3p	-5.98
277	hsa-miR-376b-3p	-5.68
278	hsa-miR-206	-5.67
279	hsa-miR-411-5p	-5.59
280	hsa-miR-146b-3p	-5.56
281	hsa-miR-127-3p	-5.50
282	hsa-miR-302d-5p	-5.39
283	hsa-miR-136-5p	-5.31
284	hsa-miR-330-3p	-4.62
285	hsa-miR-1205	-4.60
286	hsa-miR-3148	-4.52
287	hsa-miR-4536-5p	-4.52
288	hsa-miR-4670-3p	-4.52
289	hsa-miR-570-5p	-4.47
290	hsa-miR-4715-5p	-4.46
291	hsa-miR-3200-3p	-4.44
292	hsa-miR-548a-5p	-4.30
293	hsa-miR-7112-5p	-4.25
294	hsa-miR-4442	-4.24

295	hsa-miR-3117-3p	-4.23
296	hsa-miR-548ai	-4.21
297	hsa-miR-513b-5p	-4.16
298	hsa-miR-539-3p	-4.05
299	hsa-miR-376c-5p	-4.01
300	hsa-miR-642a-5p	-3.98
301	hsa-miR-187-3p	-3.97
302	hsa-miR-299-5p	-3.95
303	hsa-miR-592	-3.87
304	hsa-miR-668-3p	-3.85
305	hsa-miR-335-3p	-3.76
306	hsa-miR-371b-5p	-3.75
307	hsa-miR-543	-3.73
308	hsa-miR-338-5p	-3.63
309	hsa-miR-487a-3p	-3.54
310	hsa-miR-561-5p	-3.48
311	hsa-miR-5007-3p	-3.45
312	hsa-miR-1301-3p	-3.44
313	hsa-miR-429	-3.43
314	hsa-miR-221-3p	-3.40
315	hsa-miR-377-3p	-3.34
316	hsa-miR-1296-5p	-3.33
317	hsa-miR-146b-5p	-3.33
318	hsa-miR-132-5p	-3.32
319	hsa-miR-153-5p	-3.32
320	hsa-miR-376a-5p	-3.31
321	hsa-miR-4448	-3.27
322	hsa-miR-548ak	-3.25
323	hsa-miR-132-3p	-3.19
324	hsa-miR-628-5p	-3.19
325	hsa-miR-548aj-5p	-3.18
326	hsa-miR-216a-5p	-3.16
327	hsa-miR-325	-3.15
328	hsa-miR-6509-3p	-3.12
329	hsa-miR-200b-3p	-3.10

330	hsa-miR-4693-5p	-3.09
331	hsa-miR-421	-3.07
332	hsa-miR-149-5p	-3.03
333	hsa-miR-200b-5p	-3.02
334	hsa-miR-301a-3p	-2.95
335	hsa-miR-329-5p	-2.91
336	hsa-miR-539-5p	-2.91
337	hsa-miR-338-3p	-2.89
338	hsa-miR-593-5p	-2.89
339	hsa-miR-1250-5p	-2.88
340	hsa-miR-5699-5p	-2.87
341	hsa-miR-181c-5p	-2.84
342	hsa-miR-607	-2.84
343	hsa-miR-7-1-3p	-2.83
344	hsa-miR-377-5p	-2.80
345	hsa-miR-222-3p	-2.78
346	hsa-miR-29b-2-5p	-2.78
347	hsa-miR-200a-3p	-2.71
348	hsa-miR-4775	-2.70
349	hsa-miR-493-3p	-2.70
350	hsa-miR-224-5p	-2.69
351	hsa-miR-218-1-3p	-2.68
352	hsa-miR-4288	-2.67
353	hsa-miR-4790-5p	-2.66
354	hsa-miR-655-3p	-2.64
355	hsa-miR-1244	-2.63
356	hsa-miR-181c-3p	-2.63
357	hsa-miR-513a-3p	-2.63
358	hsa-miR-33b-5p	-2.62
359	hsa-miR-4280	-2.62
360	hsa-miR-598-3p	-2.62
361	hsa-miR-3910	-2.59
362	hsa-miR-181d-5p	-2.58
363	hsa-miR-200a-5p	-2.58
364	hsa-miR-4759	-2.57

365	hsa-miR-200c-3p	-2.56
366	hsa-miR-212-3p	-2.53
367	hsa-miR-337-3p	-2.53
368	hsa-miR-4515	-2.53
369	hsa-miR-488-5p	-2.53
370	hsa-miR-493-5p	-2.51
371	hsa-miR-628-3p	-2.50
372	hsa-miR-1251-5p	-2.49
373	hsa-miR-324-5p	-2.49
374	hsa-miR-609	-2.48
375	hsa-miR-31-5p	-2.47
376	hsa-miR-383-3p	-2.46
377	hsa-miR-221-5p	-2.45
378	hsa-miR-1267	-2.43
379	hsa-let-7e-5p	-2.42
380	hsa-miR-1262	-2.38
381	hsa-miR-4796-5p	-2.38
382	hsa-miR-552-5p	-2.38
383	hsa-miR-127-5p	-2.37
384	hsa-miR-544a	-2.37
385	hsa-miR-664a-5p	-2.36
386	hsa-miR-585-3p	-2.35
387	hsa-miR-3943	-2.34
388	hsa-miR-3139	-2.33
389	hsa-miR-1255b-5p	-2.32
390	hsa-let-7c-5p	-2.31
391	hsa-miR-1298-3p	-2.31
392	hsa-miR-3668	-2.31
393	hsa-miR-4522	-2.29
394	hsa-miR-568	-2.29
395	hsa-miR-301b-3p	-2.27
396	hsa-miR-330-5p	-2.27
397	hsa-miR-4742-5p	-2.27
398	hsa-miR-553	-2.27
399	hsa-miR-6750-5p	-2.25

400	hsa-miR-4251	-2.24
401	hsa-miR-4518	-2.24
402	hsa-miR-107	-2.23
403	hsa-miR-4260	-2.23
404	hsa-miR-1343-3p	-2.21
405	hsa-miR-1251-3p	-2.20
406	hsa-miR-296-5p	-2.20
407	hsa-miR-4476	-2.20
408	hsa-miR-496	-2.19
409	hsa-miR-1279	-2.18
410	hsa-miR-23b-5p	-2.18
411	hsa-miR-4764-3p	-2.18
412	hsa-miR-31-3p	-2.17
413	hsa-miR-3685	-2.17
414	hsa-miR-4780	-2.16
415	hsa-miR-128-3p	-2.15
416	hsa-miR-642a-3p	-2.13
417	hsa-miR-4743-3p	-2.12
418	hsa-miR-1289	-2.11
419	hsa-miR-4713-3p	-2.11
420	hsa-miR-548a-3p	-2.11
421	hsa-miR-551b-3p	-2.11
422	hsa-miR-555	-2.11
423	hsa-miR-105-5p	-2.10
424	hsa-miR-6078	-2.10
425	hsa-miR-1537-5p	-2.09
426	hsa-miR-3606-3p	-2.09
427	hsa-miR-136-3p	-2.08
428	hsa-miR-183-5p	-2.08
429	hsa-miR-3646	-2.08
430	hsa-miR-4668-5p	-2.08
431	hsa-miR-4273	-2.07
432	hsa-miR-4493	-2.07
433	hsa-miR-4519	-2.07
434	hsa-miR-1284	-2.06

435	hsa-miR-505-5p	-2.05
436	hsa-miR-29b-1-5p	-2.04
437	hsa-miR-4650-3p	-2.04
438	hsa-miR-5683	-2.04
439	hsa-miR-1273a	-2.03
440	hsa-miR-618	-2.03
441	hsa-miR-494-5p	-2.02
442	hsa-miR-191-3p	-2.01
443	hsa-miR-3115	-2.01
444	hsa-miR-4787-5p	-2.01
445	hsa-miR-491-5p	-2.01
446	hsa-miR-6867-5p	-2.01
447	hsa-miR-3613-5p	-2.00
448	hsa-miR-4798-5p	-2.00

Table S3: List of 44 target mRNAs for 41 distinctive upregulated miRNAs (includes all confidence levels)

#	**mRNA**	**Entrez Gene Name**	**mRNA Synonym(s)**	**miRNAs**	**miRNA count**
1	CCND1	cyclin D1	AI327039, B-CELL CLL/LYMPHOMA 1, bcl-1, cD1, CycD1, CYCLIN D1, G1/S-Specific Cyclin D1, PRAD1	hsa-miR-4763-3p, hsa-miR-106a-5p, hsa-miR-4664-5p, hsa-miR-6794-5p	4
2	CDH1	cadherin 1	AA960649, ARC-1, BCDS1, cadherin 1, Cadherin E, CD324, CSEIL, E-cadherin, uvomorulin	hsa-miR-4763-3p, hsa-miR-510-5p, hsa-miR-1225-3p, hsa-miR-3150a-3p, hsa-miR-7112-3p	5
3	CTNNB1	catenin beta 1	armadillo, Beta-cat, beta CATENIN, beta-Ctnn, Bfc, CATENIN beta, catenin beta 1, catenin (cadherin associated protein), beta 1, catenin (cadherin associated protein), β 1, CATENIN β, catenin β 1, CATNB, CTNN beta, CTNN β, EVR7, Mesc, MRD19, NEDSDV, β-cat, β-catenin, β-Ctnn	hsa-miR-4999-5p, hsa-miR-526b-5p	2
4	CXCL12	C-X-C motif chemokine ligand 12	chemokine (C-X-C motif) ligand 12, CXCL12 isoform 1, C-X-C motif chemokine ligand 12, hIRH, IRH, LOC105378278, PBSF, Scyb12, SDF, SDF-1alpha, SDF-1α, Stromal cell derived factor 1, TLSF, TPAR1	hsa-miR-1225-3p, hsa-miR-4653-5p, hsa-miR-455-5p, hsa-miR-6794-5p	4
5	CXCL8	C-X-C motif chemokine ligand 8	C-X-C motif chemokine ligand 8, GCP-1, IL8, LYNAP, MONAP, Monocyte-derived neutrophil chemotactic factor, NAF, NAP-1	hsa-miR-146a-5p, hsa-miR-106a-5p	2

6	ERAS	ES cell expressed Ras	Ecat5, ES cell-expressed Ras, Ha-Ras2, HRAS2, HRASP, LOC100911243, LOC682898	hsa-miR-4749-3p	1
7	GDNF	glial cell derived neurotrophic factor	Activity-dependent neurotrophic factor, AI385739, ATF, ATF1, ATF2, Glial cell derived neurotrophic factor, glial cell line derived neurotrophic factor, gndf, HFB1-GDNF, HSCR3	hsa-miR-4763-3p, hsa-miR-1292-5p, hsa-miR-146a-5p, hsa-miR-6805-5p, hsa-miR-3150a-3p, hsa-miR-4749-3p, hsa-miR-6081, hsa-miR-6813-3p, hsa-miR-6848-3p, hsa-miR-6848-5p	10
8	HNF1A	HNF1 homeobox A	AI323641, HNF1, HNF1alpha (MODY3), HNF1 homeobox A, HNF1α, HNF1α (MODY3), HNF4A, IDDM20, LFB1, TCF-1	hsa-miR-4763-3p, hsa-miR-4722-3p	2
9	KLK3	kallikrein related peptidase 3	0610007D04Rik, alpha-NGF, Egfbp2, EGF-BP A, EGF-BP C, Epidermal Growth Factor-Binding Protein Type A, gamma-NGF, hK3, HNP, KAL, KAL-B, kallikrein, kallikrein 1, kallikrein 1-related pepidase b4, kallikrein 1-related peptidase b1, kallikrein 1-related peptidase b11, kallikrein 1-related peptidase b16, kallikrein 1-related peptidase b21, kallikrein 1-related peptidase b22, kallikrein 1-related peptidase b24, kallikrein 1-related peptidase b27, kallikrein 1-related peptidase b3, kallikrein 1-related peptidase b5, kallikrein 1-related peptidase b8, kallikrein 1-related peptidase b9, kallikrein 1-related petidase b26, kallikrein related peptidase 3, Klk1, Klk11, Klk1b1, Klk1b11, Klk1b16, Klk1b21, Klk1b22, Klk1b24, Klk1b26, Klk1b27, Klk1b3, Klk1b4, Klk1b5, Klk1b6, Klk1b8, Klk1b9, Klk21, KLK21L, Klk22, Klk24, Klk27, KLK2A1, Klk5, Klk6, Klk8, Klk9, mGK-26, mK1, Nerve growth factor gamma, Nerve growth factor γ, Ngfa, Prostate-Specific Antigen, Proteinase a, Proteinase d, Proteinase f, PSA, Tissue Kallikrein, α-NGF, γ-NGF, γ-seminoprotein	hsa-miR-3150a-3p, hsa-miR-6804-5p, hsa-miR-645, hsa-miR-6758-5p	4
10	KRAS	KRAS proto-oncogene, GTPase	AI929937, CFC2, c-Ki-ras, c-Ki-ras2, c-Ki-ras p21, C-K-RAS, KI-RAS4B, Kirsten rat sarcoma viral oncogene homolog, KRAS1, KRAS2, K-RAS2A, K-RAS2B, K-RAS B, KRAS proto-oncogene, GTPase, NS, NS3, OES, p21, p21B, p21ras, RALD, RAS	hsa-miR-3150a-3p, hsa-miR-4633-5p, hsa-miR-548ae-3p, hsa-miR-548as-5p, hsa-miR-6081	5
11	LEF1	lymphoid enhancer binding factor 1	3000002B05, AI451430, lymphoid enhancer binding factor 1, TCF10, TCF7L3, TCF/LEF	hsa-miR-4664-5p	1
12	MAP2K1	mitogen-activated protein kinase kinase 1	CFC3, Mapkk1, Mek, Mek1, MEKK1, mitogen-activated protein kinase kinase 1, Mkp1	hsa-miR-6081	1

13	MAP2K2	mitogen-activated protein kinase kinase 2	AA589381, CFC4, ERK ACTIVATOR KINASE 2, MAPKK2, Mek2, mitogen-activated protein kinase kinase 2, MK2, MKK2, p45 MAPK KINASE, Prkmk2	hsa-miR-6804-5p	1
14	MAPK1	mitogen-activated protein kinase 1	9030612K14Rik, AA407128, AU018647, C78273, ERK42, ERT1, Mapk1,2, Mapk p42, mitogen-activated protein kinase 1, MITOGEN ACTIVATED protein KINASE 2, p38, p40, p40 HERAK, p41, P42, p42 Erk	hsa-miR-106a-5p	1
15	MAPK3	mitogen-activated protein kinase 3	ERT2, Esrk1, HS44KDAP, HUMKER1A, MAPK1, Mapkapk3, Mapk p44, mitogen-activated protein kinase 3, MNK1, MTAP2K, p44, p44 Erk, p44erk1, Prkm3	hsa-miR-6775-3p, hsa-miR-6805-5p, hsa-miR-6794-5p	3
16	MRAS	muscle RAS oncogene homolog	2900078C09RIK, AI326250, muscle and microspikes RAS, muscle RAS oncogene homolog, NS11, Ras3	hsa-miR-3150a-3p, hsa-miR-6794-5p, hsa-miR-6758-5p	3
17	NGF	nerve growth factor	2.5S NGF, Beta-NGF, HSAN5, nerve growth factor, Nerve growth factor, β, NGFB, Ngf beta, Ngf β, β-nerve growth factor, β-NGF	hsa-miR-4763-3p, hsa-miR-6794-5p, hsa-miR-548a	3
18	NRAS	NRAS proto-oncogene, GTPase	ALPS4, AV095280, CMNS, C-N-Ras, c-N-ras p21, DNA Segment, Chr 3, Mjeffers 1, NCMS, neuroblastoma ras oncogene, NRAS proto-oncogene, GTPase, NRASR, NS6, RAS, V-ras	hsa-miR-146a-5p, hsa-miR-4664-5p, hsa-miR-767-5p	3
19	NTF3	neurotrophin 3	AI316846, AI835689, HDNF, neurotrophin 3, NGF-2	hsa-miR-548as-5p	1
20	NTF4	neurotrophin 4	2900040K06Rik, AI462899, GLC10, GLC1O, neurotrophin 4, Neurotrophin 5, NT-4/5, NT-5, NTF5	hsa-miR-1225-3p	1
21	NTRK1	neurotrophic receptor tyrosine kinase 1	C80751, neurotrophic receptor tyrosine kinase 1, neurotrophic tyrosine kinase, receptor, type 1, p140 Trk, p140-TrkA, Trk, TRK1, TRKA, TRKA longest isoform, TRKA NGFR	hsa-miR-3616-3p, hsa-miR-6804-5p	2
22	NTRK2	neurotrophic receptor tyrosine kinase 2	AI848316, BDNF receptor, C030027L06RIK, EIEE58, GP145-TrkB, GP145-TrkB/GP95-TrkB, Gp95 Trkb, neurotrophic receptor tyrosine kinase 2, neurotrophic tyrosine kinase, receptor, type 2, Nrh2, OBHD, TRKB, TRKB long isoform, Trkb receptor	hsa-miR-3616-3p, hsa-miR-4749-3p, hsa-miR-767-5p	3
23	NTRK3	neurotrophic receptor tyrosine kinase 3	AW125844, GP145-TrkC, gp145(trkC), neurotrophic receptor tyrosine kinase 3, neurotrophic tyrosine kinase, receptor, type 3, Ntrk3 tv3, TrkC	hsa-miR-4763-3p, hsa-miR-6805-5p	2
24	PPARG	peroxisome proliferator activated receptor gamma	CIMT1, GLM1, peroxisome proliferator-activated receptor gamma, peroxisome proliferator-activated receptor γ, peroxisome proliferator activator receptor	hsa-miR-510-5p, hsa-miR-106a-5p, hsa-miR-526b-5p, hsa-miR-548as-5p, hsa-miR-5583-3p, hsa-miR-6081	6

			γ, Pparg1, PPAR gamma 3, PPARγ, PPAR γ 3, PPFP, receptor γ		
25	RALA	RAS like proto-oncogene A	3010001O15Rik, AW322615, RAS like proto-oncogene A, v-ral simian leukaemia viral oncogene A (ras related), v-ral simian leukemia viral oncogene A (ras related)	hsa-miR-548as-5p	1
26	RALB	RAS like proto-oncogene B	5730472O18Rik, dRalb, RAS like proto-oncogene B, v-ral simian leukaemia viral oncogene B, v-ral simian leukemia viral oncogene B	hsa-miR-9500	1
27	RAP1A	RAP1A, member of RAS oncogene family	AI848598, C21KG, G-22K, Krev-1, RAP1, RAP1A, member of RAS oncogene family, RAS-related protein 1a	hsa-miR-142-5p, hsa-miR-548as-5p, hsa-miR-363-3p	3
28	RAP1B	RAP1B, member of RAS oncogene family	2810443E11Rik, K-REV, RAL1B, Rap1, RAP1B, member of RAS oncogene family, RAS related protein 1b	hsa-miR-518d-3p, hsa-miR-548as-5p, hsa-miR-708-5p, hsa-miR-767-5p, hsa-miR-363-3p	5
29	RAP2A	RAP2A, member of RAS oncogene family	5830461H18RIK, K-REV, RAP2A, member of RAS oncogene family, RAS related protein 2a, RbBP-30	hsa-miR-4310, hsa-miR-4633-5p, hsa-miR-6848-5p	3
30	RAP2B	RAP2B, member of RAS oncogene family	4021402C18Rik, AA408554, RAP2B, member of RAS oncogene family	hsa-miR-105-3p, hsa-miR-6775-3p, hsa-miR-4664-5p, hsa-miR-6848-3p	4
31	RASD1	ras related dexamethasone induced 1	AGS1, DEXRAS1, MGC:26290, RAS, dexamethasone-induced 1, Ras-related, ras related dexamethasone induced 1	hsa-miR-105-3p, hsa-miR-526b-5p	2
32	RASD2	RASD family member 2	4930526B11RIK, AU045414, RASD family member 2, RASD family, member 2, Rhes, TEM-2	hsa-miR-4763-3p, hsa-miR-4664-5p, hsa-miR-3616-3p, hsa-miR-3975, hsa-miR-6081, hsa-miR-645, hsa-miR-6758-5p, hsa-miR-708-5p	8
33	RET	ret proto-oncogene	CDHR16, C-RET, PTC, RET-ELE1, RET PROTO-ONCOGENE, RET receptor	hsa-miR-510-5p	1
34	RRAS	RAS related	AI573426, p23, RAS related, related RAS viral (r-ras) oncogene, Rras predicted	hsa-miR-1225-3p, hsa-miR-3616-3p, hsa-miR-3975, hsa-miR-4749-3p	4
35	RRAS2	RAS related 2	2610016H24Rik, C86394, RAS related 2, related RAS viral (r-ras) oncogene 2, TC21	hsa-miR-3616-3p	1
36	RXRA	retinoid X receptor alpha	9530071D11RIK, LOC101928454, retinoid 10 receptor α, Retinoid X receptor alpha, Retinoid X receptor α, RXR, RXR alpha, RXRα, Rxr α 1	hsa-miR-4763-3p, hsa-miR-1225-3p, hsa-miR-6804-5p	3

37	RXRB	retinoid X receptor beta	AL023085, DAUDI6, retinoid X receptor beta, retinoid X receptor β, Rub, RXR-beta, RXR-β	hsa-miR-6804-5p	1
38	RXRG	retinoid X receptor gamma	retinoid X receptor gamma, retinoid X receptor γ, RXRC, Rxr gamma, Rxr γ	hsa-miR-3150a-3p, hsa-miR-7112-3p	2
39	SHC1	SHC adaptor protein 1	p52SHC, P66shc, Shc (46 kDa isoform), SHCA, SHC adaptor protein 1, Shc p66 isoform, src homology 2 domain-containing transforming protein C1	hsa-miR-3150a-3p, hsa-miR-3616-3p, hsa-miR-9500	3
40	TCF4	transcription factor 4	5730422P05RIK, ASP-I2, bHLHb19, E2-2, FECD3, Itf-2, PTHS, SEF2-1, SEF2-1A, SEF2-1D, transcription factor 4	hsa-miR-106a-5p, hsa-miR-6848-3p, hsa-miR-363-3p	3
41	TCF7	transcription factor 7	AI465550, TCF-1, transcription factor 7	hsa-miR-3150a-3p, hsa-miR-4664-5p, hsa-miR-4653-5p, hsa-miR-6804-5p, hsa-miR-6081, hsa-miR-645, hsa-miR-6848-5p	7
42	TCF7L1	transcription factor 7 like 1	bHLHb21, LOC100361823, TCF-3, transcription factor 7 like 1, transcription factor 7 like 1 (T cell specific, HMG box)	hsa-miR-4763-3p, hsa-miR-106a-5p, hsa-miR-6758-5p	3
43	TCF7L2	transcription factor 7 like 2	LOC683733, TCF-4, TCF4B, TCF4E, transcription factor 7 like 2, transcription factor 7 like 2, T cell specific, HMG box	hsa-miR-6805-5p, hsa-miR-4310, hsa-miR-6794-5p, hsa-miR-6804-5p, hsa-miR-6848-5p	5
44	TP53	tumor protein p53	bbl, BCC7, bfy, bhy, BMFS5, LFS1, p44, p53, P53 cellular tumour antigen, p53 tumor suppressor, transformation related protein 53, TRP53, tumor protein p53, tumour protein p53	hsa-miR-4763-3p, hsa-miR-1225-3p, hsa-miR-150-5p, hsa-miR-4664-5p	4

Table S4: List of 51 target mRNAs for 341 distinctive downregulated miRNAs (includes all confidence levels)

#	mRNA	Entrez Gene Name	mRNA Synonym(s)	miRNAs	miRNA count
1	BDNF	brain derived neurotrophic factor	ANON2, Brain derived neurotrophic factor, BULN2, OCD1	hsa-miR-206, hsa-miR-107, hsa-miR-10a-5p, hsa-miR-506-3p, hsa-miR-382-5p, hsa-miR-6867-5p, hsa-miR-499b-5p, hsa-miR-553, hsa-miR-561-5p, hsa-miR-568	10
2	BRAF	B-Raf proto-oncogene, serine/threonine kinase	9930012E13RIK, AA120551, AA387315, AA473386, AI447469, B-RAF1, B-Raf proto-oncogene, serine/threonine kinase, Braf transforming gene, C230098H17, C87398, D6Ertd631e, NS7, RAFB, RAFB1	hsa-miR-4539, hsa-miR-4803, hsa-miR-496, hsa-miR-5580-3p, hsa-miR-6853-3p	5
3	CCND1	cyclin D1	AI327039, B-CELL CLL/LYMPHOMA 1, bcl-1, cD1, CycD1, CYCLIN D1, G1/S-Specific Cyclin D1, PRAD1	hsa-let-7e-5p, hsa-miR-1229-3p, hsa-miR-3591-5p, hsa-miR-1269b, hsa-miR-136-3p, hsa-miR-1471, hsa-miR-519d-3p, hsa-miR-216b-3p, hsa-miR-221-5p, hsa-miR-302b-3p, hsa-miR-296-5p, hsa-miR-603, hsa-miR-4476, hsa-miR-4515, hsa-miR-4648, hsa-miR-6867-5p, hsa-miR-4715-3p, hsa-miR-	26

				4720-3p, hsa-miR-4796-5p, hsa-miR-488-5p, hsa-miR-520a-5p, hsa-miR-555, hsa-miR-579-5p, hsa-miR-593-5p, hsa-miR-6841-3p, hsa-miR-6878-3p	
4	CDH1	cadherin 1	AA960649, ARC-1, BCDS1, cadherin 1, Cadherin E, CD324, CSEIL, E-cadherin, uvomorulin	hsa-miR-3689b-3p, hsa-miR-1296-5p, hsa-miR-4306, hsa-miR-4782-3p, hsa-miR-3160-3p, hsa-miR-4300, hsa-miR-4711-5p, hsa-miR-544a, hsa-miR-5582-5p, hsa-miR-647	10
5	CRYGA	crystallin gamma A	CRYG5, crystallin gamma A, crystallin, gamma A, crystallin γ A, crystallin, γ A, LOC100911626, Secc, γ A crystallin	hsa-miR-568, hsa-miR-6750-5p	2
6	CTNNB 1	catenin beta 1	armadillo, Beta-cat, beta CATENIN, beta-Ctnn, Bfc, CATENIN beta, catenin beta 1, catenin (cadherin associated protein), beta 1, catenin (cadherin associated protein), β 1, CATENIN β, catenin β 1, CATNB, CTNN beta, CTNN β, EVR7, Mesc, MRD19, NEDSDV, β-cat, β-catenin, β-Ctnn	hsa-miR-200a-3p, hsa-miR-1537-5p, hsa-miR-3619-5p, hsa-miR-3119, hsa-miR-3973, hsa-miR-4251, hsa-miR-4733-5p, hsa-miR-4796-5p, hsa-miR-495-5p, hsa-miR-496, hsa-miR-5591-3p, hsa-miR-6513-5p	12
7	CXCL12	C-X-C motif chemokine ligand 12	chemokine (C-X-C motif) ligand 12, CXCL12 isoform 1, C-X-C motif chemokine ligand 12, hIRH, IRH, LOC105378278, PBSF, Scyb12, SDF, SDF-1alpha, SDF-1α, Stromal cell derived factor 1, TLSF, TPAR1	hsa-miR-1197, hsa-miR-1244, hsa-miR-1255b-5p, hsa-miR-1267, hsa-miR-3689b-3p, hsa-miR-1288-5p, hsa-miR-301a-3p, hsa-miR-6874-3p, hsa-miR-154-3p, hsa-miR-4420, hsa-miR-4306, hsa-miR-371a-3p, hsa-miR-31-5p, hsa-miR-3130-5p, hsa-miR-3157-3p, hsa-miR-3200-3p, hsa-miR-329-3p, hsa-miR-375, hsa-miR-376b-5p, hsa-miR-377-5p, hsa-miR-4318, hsa-miR-448, hsa-miR-4637, hsa-miR-4666a-3p, hsa-miR-4790-5p, hsa-miR-4798-5p, hsa-miR-5007-3p, hsa-miR-519e-3p, hsa-miR-580-3p, hsa-miR-668-3p, hsa-miR-6757-3p, hsa-miR-6841-3p	32
8	CXCL8	C-X-C motif chemokine ligand 8	C-X-C motif chemokine ligand 8, GCP-1, IL8, LYNAP, MONAP, Monocyte-derived neutrophil chemotactic factor, NAF, NAP-1	hsa-let-7e-5p, hsa-miR-1207-3p, hsa-miR-506-3p, hsa-miR-146b-5p, hsa-miR-6874-3p, hsa-miR-154-5p, hsa-miR-519d-3p, hsa-miR-4420, hsa-miR-4306, hsa-miR-1973, hsa-miR-221-5p, hsa-miR-302b-3p, hsa-miR-3126-3p, hsa-miR-4439, hsa-miR-573, hsa-miR-3674, hsa-miR-369-3p, hsa-miR-376a-5p, hsa-miR-4272, hsa-miR-4282, hsa-miR-4318, hsa-miR-4445-5p, hsa-miR-4670-3p, hsa-miR-4699-3p, hsa-miR-4799-3p, hsa-miR-6744-3p, hsa-miR-5706, hsa-miR-4795-5p, hsa-miR-493-5p, hsa-miR-5582-5p, hsa-miR-5692b, hsa-miR-587, hsa-miR-6878-3p	33
9	ERAS	ES cell expressed Ras	Ecat5, ES cell-expressed Ras, Ha-Ras2, HRAS2, HRASP, LOC100911243, LOC682898	hsa-miR-3130-5p, hsa-miR-543, hsa-miR-5582-5p	3

10	GDNF	glial cell derived neurotrophic factor	Activity-dependent neurotrophic factor, AI385739, ATF, ATF1, ATF2, Glial cell derived neurotrophic factor, glial cell line derived neurotrophic factor, gndf, HFB1-GDNF, HSCR3	hsa-miR-4319, hsa-miR-1298-3p, hsa-miR-1343-3p, hsa-miR-146b-5p, hsa-miR-20b-3p, hsa-miR-215-3p, hsa-miR-218-1-3p, hsa-miR-3115, hsa-miR-3165, hsa-miR-3191-5p, hsa-miR-330-5p, hsa-miR-3652, hsa-miR-3650, hsa-miR-4300, hsa-miR-4493, hsa-miR-4668-5p, hsa-miR-4731-5p, hsa-miR-548ag, hsa-miR-593-5p, hsa-miR-604, hsa-miR-647, hsa-miR-668-3p, hsa-miR-6757-3p	23
11	HNF1A	HNF1 homeobox A	AI323641, HNF1, HNF1alpha (MODY3), HNF1 homeobox A, HNF1α, HNF1α (MODY3), HNF4A, IDDM20, LFB1, TCF-1	hsa-miR-1343-3p, hsa-miR-4745-3p, hsa-miR-3619-5p, hsa-miR-221-5p, hsa-miR-381-5p, hsa-miR-4260, hsa-miR-4493, hsa-miR-4648, hsa-miR-4711-5p, hsa-miR-5699-5p, hsa-miR-604, hsa-miR-6501-3p	12
12	HRAS	HRas proto-oncogene, GTPase	C-BAS/HAS, C-HA-RAS, C-H-RAS, c-Ki-ras, c-K-ras, c-rasHa, CTLO, HAMSV, HA-RAS, Harvey-ras, Harvey rat sarcoma virus oncogene, Hras-1, H-RASIDX, HRas proto-oncogene, GTPase, Ki-Ras, KRAS2, RAS, RASH1, RAS HA, RASK2, V-H-RAS	hsa-miR-1199-3p, hsa-miR-1256, hsa-miR-3689b-3p, hsa-miR-1301-3p, hsa-miR-23b-5p, hsa-miR-4300, hsa-miR-4318, hsa-miR-4446-3p, hsa-miR-4693-5p, hsa-miR-4731-5p, hsa-miR-585-3p, hsa-miR-6513-5p, hsa-miR-6841-3p, hsa-miR-4756-5p	14
13	KLK3	kallikrein related peptidase 3	0610007D04Rik, alpha-NGF, Egfbp2, EGF-BP A, EGF-BP C, Epidermal Growth Factor-Binding Protein Type A, gamma-NGF, hK3, HNP, KAL, KAL-B, kallikrein, kallikrein 1, kallikrein 1-related pepidase b4, kallikrein 1-related peptidase b1, kallikrein 1-related peptidase b11, kallikrein 1-related peptidase b16, kallikrein 1-related peptidase b21, kallikrein 1-related peptidase b22, kallikrein 1-related peptidase b24, kallikrein 1-related peptidase b27, kallikrein 1-related peptidase b3, kallikrein 1-related peptidase b5, kallikrein 1-related peptidase b8, kallikrein 1-related peptidase b9, kallikrein 1-related petidase b26, kallikrein related peptidase 3, Klk1, Klk11, Klk1b1, Klk1b11, Klk1b16, Klk1b21, Klk1b22, Klk1b24, Klk1b26, Klk1b27, Klk1b3, Klk1b4, Klk1b5, Klk1b6, Klk1b8,	hsa-miR-1197, hsa-miR-190b, hsa-miR-384, hsa-miR-494-5p, hsa-miR-4288, hsa-miR-4639-3p, hsa-miR-1271-3p, hsa-miR-550b-2-5p, hsa-miR-668-3p, hsa-miR-6841-3p	10

			Klk1b9, Klk21, KLK21L, Klk22, Klk24, Klk27, KLK2A1, Klk5, Klk6, Klk8, Klk9, mGK-26, mK1, Nerve growth factor gamma, Nerve growth factor γ, Ngfa, Prostate-Specific Antigen, Proteinase a, Proteinase d, Proteinase f, PSA, Tissue Kallikrein, α-NGF, γ-NGF, γ-seminoprotein		
14	KRAS	KRAS proto-oncogene, GTPase	AI929937, CFC2, c-Ki-ras, c-Ki-ras2, c-Ki-ras p21, C-K-RAS, KI-RAS4B, Kirsten rat sarcoma viral oncogene homolog, KRAS1, KRAS2, K-RAS2A, K-RAS2B, K-RAS B, KRAS proto-oncogene, GTPase, NS, NS3, OES, p21, p21B, p21ras, RALD, RAS	hsa-let-7e-5p, hsa-miR-206, hsa-miR-105-5p, hsa-miR-3591-5p, hsa-miR-1256, hsa-miR-132-3p, hsa-miR-181c-5p, hsa-miR-181c-3p, hsa-miR-215-3p, hsa-miR-216b-3p, hsa-miR-3130-5p, hsa-miR-3136-3p, hsa-miR-3148, hsa-miR-330-5p, hsa-miR-603, hsa-miR-376c-3p, hsa-miR-377-3p, hsa-miR-383-3p, hsa-miR-3910, hsa-miR-4323, hsa-miR-4424, hsa-miR-4445-5p, hsa-miR-4519, hsa-miR-4678, hsa-miR-4694-3p, hsa-miR-548aj-3p, hsa-miR-548ag, hsa-miR-548a-5p, hsa-miR-5582-5p, hsa-miR-5699-5p, hsa-miR-6500-3p, hsa-miR-6509-3p, hsa-miR-6509-5p	33
15	LEF1	lymphoid enhancer binding factor 1	3000002B05, AI451430, lymphoid enhancer binding factor 1, TCF10, TCF7L3, TCF/LEF	hsa-miR-1251-5p, hsa-miR-1258, hsa-miR-4782-3p, hsa-miR-302b-3p, hsa-miR-3119, hsa-miR-381-3p, hsa-miR-412-5p, hsa-miR-4446-3p, hsa-miR-4506, hsa-miR-4519, hsa-miR-4645-3p, hsa-miR-4697-3p, hsa-miR-4799-3p, hsa-miR-4742-5p, hsa-miR-621, hsa-miR-6841-3p	16
16	MAP2K 1	mitogen-activated protein kinase kinase 1	CFC3, Mapkk1, Mek, Mek1, MEKK1, mitogen-activated protein kinase kinase 1, Mkp1	hsa-miR-181c-5p, hsa-miR-3136-3p, hsa-miR-376a-3p, hsa-miR-3943, hsa-miR-4278, hsa-miR-4323, hsa-miR-4639-3p, hsa-miR-4645-3p, hsa-miR-4781-3p, hsa-miR-664a-5p, hsa-miR-485-3p, hsa-miR-5582-5p, hsa-miR-5699-5p, hsa-miR-7106-3p, hsa-miR-7112-5p	15
17	MAP2K 2	mitogen-activated protein kinase kinase 2	AA589381, CFC4, ERK ACTIVATOR KINASE 2, MAPKK2, Mek2, mitogen-activated protein kinase kinase 2, MK2, MKK2, p45 MAPK KINASE, Prkmk2	hsa-miR-1207-3p, hsa-miR-1250-5p, hsa-miR-2052, hsa-miR-4316, hsa-miR-579-5p	5
18	MAPK1	mitogen-activated protein kinase 1	9030612K14Rik, AA407128, AU018647, C78273, ERK42, ERT1, Mapk1,2, Mapk p42, mitogen-activated protein kinase 1, MITOGEN ACTIVATED protein KINASE 2, p38, p40, p40 HERAK, p41, P42, p42 Erk	hsa-miR-1229-3p, hsa-miR-1269b, hsa-miR-1288-3p, hsa-miR-301a-3p, hsa-miR-132-3p, hsa-miR-1537-5p, hsa-miR-519d-3p, hsa-miR-208b-5p, hsa-miR-3619-5p, hsa-miR-6129, hsa-miR-3191-5p, hsa-miR-330-5p, hsa-miR-573, hsa-miR-4526, hsa-miR-381-5p, hsa-miR-409-5p, hsa-miR-4303, hsa-miR-4692, hsa-miR-4515, hsa-miR-4645-3p, hsa-miR-491-5p, hsa-miR-585-3p, hsa-miR-621, hsa-miR-628-5p	24

19	MAPK3	mitogen-activated protein kinase 3	ERT2, Esrk1, HS44KDAP, HUMKER1A, MAPK1, Mapkapk3, Mapk p44, mitogen-activated protein kinase 3, MNK1, MTAP2K, p44, p44 Erk, p44erk1, Prkm3	hsa-miR-129-2-3p, hsa-miR-132-3p, hsa-miR-4745-3p, hsa-miR-3619-5p, hsa-miR-3652, hsa-miR-3672, hsa-miR-4448, hsa-miR-4451, hsa-miR-4536-5p, hsa-miR-4731-5p, hsa-miR-491-5p, hsa-miR-4756-5p	12
20	MRAS	muscle RAS oncogene homolog	2900078C09RIK, AI326250, muscle and microspikes RAS, muscle RAS oncogene homolog, NS11, Ras3	hsa-miR-297, hsa-miR-29b-2-5p, hsa-miR-3668, hsa-miR-376b-5p, hsa-miR-4276, hsa-miR-491-5p, hsa-miR-6508-3p, hsa-miR-6854-5p, hsa-miR-555, hsa-miR-7106-3p	10
21	MYC	MYC proto-oncogene, bHLH transcription factor	AU016757, bHLHe39, C-MYC-P64, CMYC, MRTL, Myc2, MYC proto-oncogene, bHLH transcription factor, MYCC, myelocytomatosis oncogene	hsa-let-7e-5p, hsa-miR-33b-5p, hsa-miR-377-5p, hsa-miR-4432, hsa-miR-4699-3p, hsa-miR-494-3p, hsa-miR-5584-3p, hsa-miR-655-5p	8
22	NGF	nerve growth factor	2.5S NGF, Beta-NGF, HSAN5, nerve growth factor, Nerve growth factor, β, NGFB, Ngf beta, Ngf β, β-nerve growth factor, β-NGF	hsa-let-7e-5p, hsa-miR-4280, hsa-miR-548a-3p, hsa-miR-4756-5p	4
23	NRAS	NRAS proto-oncogene, GTPase	ALPS4, AV095280, CMNS, C-N-Ras, c-N-ras p21, DNA Segment, Chr 3, Mjeffers 1, NCMS, neuroblastoma ras oncogene, NRAS proto-oncogene, GTPase, NRASR, NS6, RAS, V-ras	hsa-let-7e-5p, hsa-miR-506-3p, hsa-miR-1245b-3p, hsa-miR-1301-3p, hsa-miR-146b-5p, hsa-miR-6874-3p, hsa-miR-4420, hsa-miR-218-1-3p, hsa-miR-371b-5p, hsa-miR-372-5p, hsa-miR-3191-5p, hsa-miR-3650, hsa-miR-3672, hsa-miR-421, hsa-miR-4251, hsa-miR-4697-3p, hsa-miR-4731-5p, hsa-miR-485-3p, hsa-miR-598-3p, hsa-miR-6509-3p, hsa-miR-6509-5p, hsa-miR-6761-5p	22
24	NTF3	neurotrophin 3	AI316846, AI835689, HDNF, neurotrophin 3, NGF-2	hsa-miR-132-3p, hsa-miR-429, hsa-miR-221-3p, hsa-miR-338-5p, hsa-miR-448, hsa-miR-4670-3p, hsa-miR-4672, hsa-miR-548a-5p, hsa-miR-5683, hsa-miR-5692b, hsa-miR-628-5p	11
25	NTF4	neurotrophin 4	2900040K06Rik, AI462899, GLC10, GLC1O, neurotrophin 4, Neurotrophin 5, NT-4/5, NT-5, NTF5	hsa-miR-187-3p, hsa-miR-4727-5p, hsa-miR-134-5p, hsa-miR-4486, hsa-miR-585-3p	5
26	NTRK1	neurotrophic receptor tyrosine kinase 1	C80751, neurotrophic receptor tyrosine kinase 1, neurotrophic tyrosine kinase, receptor, type 1, p140 Trk, p140-TrkA, Trk, TRK1, TRKA, TRKA longest isoform, TRKA NGFR	hsa-miR-1180-3p, hsa-miR-31-3p, hsa-miR-3200-3p, hsa-miR-3943, hsa-miR-4300, hsa-miR-4731-5p, hsa-miR-4757-3p	7
27	NTRK2	neurotrophic receptor tyrosine kinase 2	AI848316, BDNF receptor, C030027L06RIK, EIEE58, GP145-TrkB, GP145-	hsa-miR-107, hsa-miR-1205, hsa-miR-1178-5p, hsa-miR-1263, hsa-miR-1266-3p, hsa-miR-1269b, hsa-miR-4306, hsa-miR-19b-2-	27

			TrkB/GP95-TrkB, Gp95 Trkb, neurotrophic receptor tyrosine kinase 2, neurotrophic tyrosine kinase, receptor, type 2, Nrh2, OBHD, TRKB, TRKB long isoform, Trkb receptor	5p, hsa-miR-216b-3p, hsa-miR-221-5p, hsa-miR-3191-5p, hsa-miR-3200-3p, hsa-miR-4264, hsa-miR-4266, hsa-miR-4276, hsa-miR-4278, hsa-miR-4323, hsa-miR-4515, hsa-miR-4645-3p, hsa-miR-4666a-5p, hsa-miR-6867-5p, hsa-miR-4694-3p, hsa-miR-6744-3p, hsa-miR-544a, hsa-miR-6511b-5p, hsa-miR-6841-3p, hsa-miR-6854-3p	
28	NTRK3	neurotrophic receptor tyrosine kinase 3	AW125844, GP145-TrkC, gp145(trkC), neurotrophic receptor tyrosine kinase 3, neurotrophic tyrosine kinase, receptor, type 3, Ntrk3 tv3, TrkC	hsa-miR-3689b-3p, hsa-miR-3681-3p, hsa-miR-3121-5p, hsa-miR-494-5p, hsa-miR-4276, hsa-miR-4303, hsa-miR-4316, hsa-miR-4742-5p, hsa-miR-4795-5p, hsa-miR-485-3p, hsa-miR-491-5p, hsa-miR-4756-5p	12
29	PPARG	peroxisome proliferator activated receptor gamma	CIMT1, GLM1, peroxisome proliferator-activated receptor gamma, peroxisome proliferator-activated receptor γ, peroxisome proliferator activator receptor γ, Pparg1, PPAR gamma 3, PPARγ, PPAR γ 3, PPFP, receptor γ	hsa-miR-1207-3p, hsa-miR-4518, hsa-miR-3681-3p, hsa-miR-301a-3p, hsa-miR-519d-3p, hsa-miR-215-3p, hsa-miR-494-5p, hsa-miR-4781-3p, hsa-miR-513c-3p, hsa-miR-548a-5p, hsa-miR-654-3p	11
30	RALA	RAS like proto-oncogene A	3010001O15Rik, AW322615, RAS like proto-oncogene A, v-ral simian leukaemia viral oncogene A (ras related), v-ral simian leukemia viral oncogene A (ras related)	hsa-miR-183-5p, hsa-miR-3117-3p, hsa-miR-329-3p, hsa-miR-3689e, hsa-miR-3910, hsa-miR-4697-3p, hsa-miR-548a-5p	7
31	RALB	RAS like proto-oncogene B	5730472O18Rik, dRalb, RAS like proto-oncogene B, v-ral simian leukaemia viral oncogene B, v-ral simian leukemia viral oncogene B	hsa-let-7e-5p, hsa-miR-3689b-3p, hsa-miR-1288-5p, hsa-miR-1298-3p, hsa-miR-149-5p, hsa-miR-181c-3p, hsa-miR-190b, hsa-miR-20b-3p, hsa-miR-4727-5p, hsa-miR-3126-3p, hsa-miR-3160-3p, hsa-miR-3165, hsa-miR-4266, hsa-miR-4288, hsa-miR-433-3p, hsa-miR-4448, hsa-miR-4692, hsa-miR-4662a-5p, hsa-miR-6867-5p, hsa-miR-4712-3p, hsa-miR-4735-5p, hsa-miR-664a-5p, hsa-miR-5007-3p, hsa-miR-5704, hsa-miR-592, hsa-miR-593-5p, hsa-miR-6501-3p	27
32	RAP1A	RAP1A, member of RAS oncogene family	AI848598, C21KG, G-22K, Krev-1, RAP1, RAP1A, member of RAS oncogene family, RAS-related protein 1a	hsa-miR-1245b-3p, hsa-miR-5590-3p, hsa-miR-149-5p, hsa-miR-1537-5p, hsa-miR-3126-3p, hsa-miR-337-3p, hsa-miR-573, hsa-miR-4526, hsa-miR-433-3p, hsa-miR-4471, hsa-miR-4645-3p, hsa-miR-4672, hsa-miR-4733-5p, hsa-miR-4766-3p, hsa-miR-664a-5p, hsa-miR-548a-5p, hsa-miR-5579-3p, hsa-miR-568, hsa-miR-5683, hsa-miR-587, hsa-miR-6501-3p, hsa-miR-6750-5p, hsa-miR-4756-5p, hsa-miR-367-3p	24
33	RAP1B	RAP1B, member of RAS oncogene family	2810443E11Rik, K-REV, RAL1B, Rap1, RAP1B, member of RAS oncogene family, RAS related protein 1b	hsa-miR-1185-1-3p, hsa-miR-206, hsa-miR-1244, hsa-miR-1245b-5p, hsa-miR-1256, hsa-miR-1267, hsa-miR-1269b, hsa-miR-3681-3p, hsa-miR-129-2-3p, hsa-miR-149-5p, hsa-miR-429, hsa-miR-3117-3p, hsa-	39

				miR-3119, hsa-miR-3165, hsa-miR-323b-3p, hsa-miR-329-5p, hsa-miR-330-3p, hsa-miR-3910, hsa-miR-3973, hsa-miR-4318, hsa-miR-4666a-5p, hsa-miR-4693-5p, hsa-miR-588, hsa-miR-4703-3p, hsa-miR-4742-5p, hsa-miR-4759, hsa-miR-4781-3p, hsa-miR-4796-5p, hsa-miR-485-3p, hsa-miR-494-3p, hsa-miR-518c-3p, hsa-miR-520d-5p, hsa-miR-548a-5p, hsa-miR-5580-3p, hsa-miR-5704, hsa-miR-6501-3p, hsa-miR-3139, hsa-miR-7106-3p, hsa-miR-367-3p	
34	RAP2A	RAP2A, member of RAS oncogene family	5830461H18RIK, K-REV, RAP2A, member of RAS oncogene family, RAS related protein 2a, RbBP-30	hsa-miR-1205, hsa-miR-10a-5p, hsa-miR-1185-5p, hsa-miR-1266-3p, hsa-miR-3681-3p, hsa-miR-129-2-3p, hsa-miR-6502-5p, hsa-miR-181c-5p, hsa-miR-200b-5p, hsa-miR-20b-3p, hsa-miR-216b-5p, hsa-miR-224-3p, hsa-miR-29b-1-5p, hsa-miR-29b-2-5p, hsa-miR-4727-5p, hsa-miR-3130-5p, hsa-miR-3191-5p, hsa-miR-33b-5p, hsa-miR-330-5p, hsa-miR-335-5p, hsa-miR-4526, hsa-miR-380-3p, hsa-miR-3910, hsa-miR-4318, hsa-miR-4424, hsa-miR-4446-3p, hsa-miR-4493, hsa-miR-4495, hsa-miR-4666a-5p, hsa-miR-4672, hsa-miR-4711-5p, hsa-miR-4719, hsa-miR-4724-3p, hsa-miR-4733-5p, hsa-miR-4761-5p, hsa-miR-4764-3p, hsa-miR-4780, hsa-miR-485-3p, hsa-miR-488-5p, hsa-miR-495-5p, hsa-miR-544a, hsa-miR-5590-5p, hsa-miR-561-5p, hsa-miR-568, hsa-miR-587, hsa-miR-642a-3p, hsa-miR-642a-5p, hsa-miR-6509-3p, hsa-miR-655-5p, hsa-miR-6738-3p, hsa-miR-7-1-3p	51
35	RAP2B	RAP2B, member of RAS oncogene family	4021402C18Rik, AA408554, RAP2B, member of RAS oncogene family	hsa-miR-506-3p, hsa-miR-132-3p, hsa-miR-153-3p, hsa-miR-221-5p, hsa-miR-4709-3p, hsa-miR-3157-3p, hsa-miR-329-3p, hsa-miR-330-3p, hsa-miR-337-5p, hsa-miR-603, hsa-miR-4442, hsa-miR-4300, hsa-miR-4448, hsa-miR-4668-5p, hsa-miR-4711-5p, hsa-miR-4731-5p, hsa-miR-4795-5p, hsa-miR-488-3p, hsa-miR-553, hsa-miR-5681a, hsa-miR-593-5p, hsa-miR-7112-5p	22
36	RAS			hsa-let-7e-5p	1
37	RASD1	ras related dexamethasone induced 1	AGS1, DEXRAS1, MGC:26290, RAS, dexamethasone-induced 1, Ras-related, ras related dexamethasone induced 1	hsa-miR-4745-3p, hsa-miR-3200-3p, hsa-miR-4283, hsa-miR-4486, hsa-miR-4731-5p, hsa-miR-4787-5p, hsa-miR-491-5p, hsa-miR-604	8
38	RASD2	RASD family member 2	4930526B11RIK, AU045414, RASD family member 2, RASD family, member 2, Rhes, TEM-2	hsa-miR-1197, hsa-miR-506-3p, hsa-miR-4319, hsa-miR-127-3p, hsa-miR-1296-5p, hsa-miR-4306, hsa-miR-6129, hsa-miR-3115, hsa-miR-3126-3p, hsa-miR-3160-3p, hsa-miR-3177-5p, hsa-miR-4266, hsa-miR-4300, hsa-miR-4493, hsa-miR-4692, hsa-miR-6867-5p, hsa-miR-4799-3p, hsa-miR-4731-5p, hsa-miR-485-5p, hsa-miR-491-5p, hsa-miR-1271-3p, hsa-miR-585-3p, hsa-miR-609, hsa-miR-3139, hsa-miR-7112-5p	25

39	RET	ret proto-oncogene	CDHR16, C-RET, PTC, RET-ELE1, RET PROTO-ONCOGENE, RET receptor	hsa-miR-107, hsa-miR-3681-3p, hsa-miR-4709-3p, hsa-miR-4526, hsa-miR-655-3p, hsa-miR-3943, hsa-miR-4672, hsa-miR-544a, hsa-miR-561-5p	9
40	RRAS	RAS related	AI573426, p23, RAS related, related RAS viral (r-ras) oncogene, Rras predicted	hsa-miR-1185-1-3p, hsa-miR-506-3p, hsa-miR-3681-3p, hsa-miR-1343-3p, hsa-miR-18b-5p, hsa-miR-4519, hsa-miR-485-5p, hsa-miR-491-5p, hsa-miR-5582-5p	9
41	RRAS2	RAS related 2	2610016H24Rik, C86394, RAS related 2, related RAS viral (r-ras) oncogene 2, TC21	hsa-miR-127-5p, hsa-miR-4306, hsa-miR-221-5p, hsa-miR-3674, hsa-miR-376a-5p, hsa-miR-382-5p, hsa-miR-4251, hsa-miR-4276, hsa-miR-4759, hsa-miR-5094, hsa-miR-5579-3p, hsa-miR-654-3p	12
42	RXRA	retinoid X receptor alpha	9530071D11RIK, LOC101928454, retinoid 10 receptor α, Retinoid X receptor alpha, Retinoid X receptor α, RXR, RXR alpha, RXRα, Rxr α 1	hsa-miR-4323, hsa-miR-4692, hsa-miR-5699-5p, hsa-miR-604, hsa-miR-6501-3p, hsa-miR-7112-5p	6
43	RXRB	retinoid X receptor beta	AL023085, DAUDI6, retinoid X receptor beta, retinoid X receptor β, Rub, RXR-beta, RXR-β	hsa-miR-1229-3p, hsa-miR-1289, hsa-miR-1298-3p, hsa-miR-296-5p, hsa-miR-4727-5p, hsa-miR-4731-5p, hsa-miR-5699-5p, hsa-miR-6738-3p	8
44	RXRG	retinoid X receptor gamma	retinoid X receptor gamma, retinoid X receptor γ, RXRC, Rxr gamma, Rxr γ	hsa-miR-3689b-3p, hsa-miR-1284, hsa-miR-297, hsa-miR-377-3p, hsa-miR-4260, hsa-miR-4476, hsa-miR-491-5p, hsa-miR-556-3p	8
45	SHC1	SHC adaptor protein 1	p52SHC, P66shc, Shc (46 kDa isoform), SHCA, SHC adaptor protein 1, Shc p66 isoform, src homology 2 domain-containing transforming protein C1	hsa-miR-1207-3p, hsa-miR-1229-3p, hsa-miR-506-3p, hsa-miR-153-3p, hsa-miR-5706, hsa-miR-5582-5p	6
46	TCF3	transcription factor 3	AA408400, AGM8, AW209082, bHLHb21, E12/E47, E2-5, KA1, LOC100364490, LOC688265, p75, Pan1, Pan2, TCF3 isoform 1, Tcfe2a, TRANSCRIPTION FACTOR 3, VDIR	hsa-miR-506-3p, hsa-miR-190b, hsa-miR-297, hsa-miR-324-5p, hsa-miR-3650, hsa-miR-4526, hsa-miR-376b-5p, hsa-miR-3910, hsa-miR-6867-5p, hsa-miR-4790-5p, hsa-miR-5582-5p, hsa-miR-3129-3p, hsa-miR-628-5p	13
47	TCF4	transcription factor 4	5730422P05RIK, ASP-I2, bHLHb19, E2-2, FECD3, Itf-2, PTHS, SEF2-1, SEF2-1A, SEF2-1D, transcription factor 4	hsa-miR-4256, hsa-miR-506-3p, hsa-miR-1251-3p, hsa-miR-1256, hsa-miR-1278, hsa-miR-1301-3p, hsa-miR-301a-3p, hsa-miR-153-3p, hsa-miR-519d-3p, hsa-miR-183-5p, hsa-miR-190b, hsa-miR-3115, hsa-miR-3117-3p, hsa-miR-3672, hsa-miR-379-5p, hsa-miR-4795-5p, hsa-miR-4796-5p, hsa-miR-485-3p, hsa-miR-499b-5p, hsa-miR-5582-5p, hsa-miR-5590-5p, hsa-miR-5699-5p, hsa-miR-7-1-3p, hsa-miR-367-3p, hsa-miR-628-5p	25
48	TCF7	transcription factor 7	AI465550, TCF-1, transcription factor 7	hsa-miR-1184, hsa-miR-1207-3p, hsa-miR-1245b-5p, hsa-miR-4319, hsa-miR-6736-5p, hsa-miR-4518, hsa-miR-127-5p, hsa-miR-3689b-3p, hsa-miR-1288-3p, hsa-miR-129-2-	36

				3p, hsa-miR-1343-3p, hsa-miR-4745-3p, hsa-miR-215-3p, hsa-miR-31-5p, hsa-miR-3652, hsa-miR-376a-3p, hsa-miR-4251, hsa-miR-4506, hsa-miR-4692, hsa-miR-4648, hsa-miR-4697-3p, hsa-miR-4718, hsa-miR-4731-5p, hsa-miR-4757-3p, hsa-miR-664a-5p, hsa-miR-491-5p, hsa-miR-6508-3p, hsa-miR-1271-3p, hsa-miR-5704, hsa-miR-642a-3p, hsa-miR-6499-3p, hsa-miR-6511b-5p, hsa-miR-655-5p, hsa-miR-6761-5p, hsa-miR-6841-3p, hsa-miR-4756-5p	
49	TCF7L1	transcription factor 7 like 1	bHLHb21, LOC100361823, TCF-3, transcription factor 7 like 1, transcription factor 7 like 1 (T cell specific, HMG box)	hsa-miR-3689b-3p, hsa-miR-132-3p, hsa-miR-519d-3p, hsa-miR-6129, hsa-miR-330-5p, hsa-miR-409-5p, hsa-miR-431-5p, hsa-miR-4731-5p, hsa-miR-4787-5p, hsa-miR-519b-3p, hsa-miR-6750-5p	11
50	TCF7L2	transcription factor 7 like 2	LOC683733, TCF-4, TCF4B, TCF4E, transcription factor 7 like 2, transcription factor 7 like 2, T cell specific, HMG box	hsa-miR-1185-1-3p, hsa-miR-206, hsa-miR-1207-3p, hsa-miR-1258, hsa-miR-1267, hsa-miR-181c-5p, hsa-miR-4420, hsa-miR-183-5p, hsa-miR-218-1-3p, hsa-miR-221-3p, hsa-miR-24-1-5p, hsa-miR-297, hsa-miR-6129, hsa-miR-3119, hsa-miR-3135a, hsa-miR-3136-3p, hsa-miR-3177-5p, hsa-miR-329-5p, hsa-miR-337-5p, hsa-miR-655-3p, hsa-miR-377-3p, hsa-miR-380-3p, hsa-miR-3973, hsa-miR-4300, hsa-miR-4446-3p, hsa-miR-4451, hsa-miR-4506, hsa-miR-4650-3p, hsa-miR-4668-5p, hsa-miR-4789-5p, hsa-miR-4731-5p, hsa-miR-4761-5p, hsa-miR-5706, hsa-miR-485-3p, hsa-miR-493-5p, hsa-miR-494-3p, hsa-miR-495-5p, hsa-miR-569, hsa-miR-628-3p, hsa-miR-651-3p	40
51	TP53	tumor protein p53	bbl, BCC7, bfy, bhy, BMFS5, LFS1, p44, p53, P53 cellular tumour antigen, p53 tumor suppressor, transformation related protein 53, TRP53, tumor protein p53, tumour protein p53	hsa-let-7e-5p, hsa-miR-224-5p, hsa-miR-4319, hsa-miR-1269b, hsa-miR-3689b-3p, hsa-miR-181c-3p, hsa-miR-296-5p, hsa-miR-6129, hsa-miR-3165, hsa-miR-338-3p, hsa-miR-3652, hsa-miR-376a-3p, hsa-miR-379-5p, hsa-miR-494-5p, hsa-miR-421, hsa-miR-433-5p, hsa-miR-6855-3p, hsa-miR-4692, hsa-miR-4697-3p, hsa-miR-4715-5p, hsa-miR-4742-5p, hsa-miR-4795-5p, hsa-miR-485-5p, hsa-miR-488-5p, hsa-miR-491-5p, hsa-miR-562, hsa-miR-6078	27

Table S5: Relationships between the molecules (miRNAs and mRNAs) within the Th1 pathway

From Molecule(s)	Relationship Type	To Molecule(s)
CD40	expression	CD40
CD40	expression	IL10
CD40	expression	IL6
CD40	localization	IL10
CD40	localization	IL6
CD40	phosphorylation	STAT3

IFNA1/IFNA13	phosphorylation	STAT3
IFNAR1	expression	IFNAR1
IFNAR1	expression	miR-1225-3p (miRNAs w/seed GAGCCCC)
IFNAR1	phosphorylation	STAT3
IFNAR1	ubiquitination	IFNAR1
IL10	activation	STAT3
IL10	expression	CD40
IL10	expression	IL10
IL10	expression	IL6
IL10	localization	IL6
IL10	phosphorylation	STAT3
IL10	transcription	IL10
IL6	activation	JAK1
IL6	activation	STAT3
IL6	expression	IL6
IL6	expression	STAT3
IL6	localization	STAT3
IL6	phosphorylation	JAK1
IL6	phosphorylation	STAT3
IL6	regulation of binding	JAK1
IL6	regulation of binding	STAT3
IL6	transcription	IL6
IL6	translocation	STAT3
JAK1	activation	STAT3
JAK1	expression	CD40
JAK1	expression	IL10
JAK1	localization	IL10
JAK1	localization	IL6
JAK1	phosphorylation	STAT3
JAK1	protein-protein interactions	STAT3
PIK3R1	expression	PIK3R1
STAT3	expression	CD40
STAT3	expression	IL10
STAT3	expression	IL6
STAT3	expression	STAT3

STAT3	expression	miR-181a-5p (and other miRNAs w/seed ACAUUCA)
STAT3	localization	IL10
STAT3	localization	IL6
STAT3	localization	STAT3
STAT3	phosphorylation	STAT3
STAT3	protein-DNA interactions	IL10
STAT3	protein-DNA interactions	IL6
STAT3	protein-DNA interactions	STAT3
STAT3	protein-protein interactions	JAK1
STAT3	regulation of binding	STAT3
STAT3	transcription	STAT3
miR-146a-5p (and other miRNAs w/seed GAGAACU)	RNA-RNA interactions: microRNA targeting	CD40
miR-146a-5p (and other miRNAs w/seed GAGAACU)	RNA-RNA interactions: microRNA targeting	IFNA1/IFNA13
miR-146a-5p (and other miRNAs w/seed GAGAACU)	RNA-RNA interactions: microRNA targeting	IL10
miR-146a-5p (and other miRNAs w/seed GAGAACU)	RNA-RNA interactions: microRNA targeting	IL12RB2
miR-146a-5p (and other miRNAs w/seed GAGAACU)	expression	CD40
miR-146a-5p (and other miRNAs w/seed GAGAACU)	expression	IFNA1/IFNA13
miR-146a-5p (and other miRNAs w/seed GAGAACU)	expression	IL10
miR-146a-5p (and other miRNAs w/seed GAGAACU)	expression	IL12RB2
miR-146a-5p (and other miRNAs w/seed GAGAACU)	expression	TBX21
miR-221-3p (and other miRNAs w/seed GCUACAU)	RNA-RNA interactions: microRNA targeting	PIK3R1
miR-221-3p (and other miRNAs w/seed GCUACAU)	expression	PIK3R1
miR-495-3p (and other miRNAs w/seed AACAAAC)	expression	IL6
miR-543-3p (and other miRNAs w/seed AACAUUC)	expression	IL6
mir-146	expression	IL6
mir-146	expression	miR-146a-5p (and other miRNAs w/seed GAGAACU)

Table S6: Relationships between the molecules (miRNAs and mRNAs) within the Th2 pathway.

From Molecule(s)	Relationship Type	To Molecule(s)
CD40	expression	CD40
CD40	expression	IL10
CD40	localization	IL10
GFI1	expression	miR-291a-3p (and other miRNAs w/seed AAGUGCU)
GFI1	molecular cleavage	GFI1
GFI1	regulation of binding	GFI1
IL10	expression	CD40
IL10	expression	IL10
IL10	transcription	IL10
JAK1	expression	CD40
JAK1	expression	IL10
JAK1	localization	IL10
JUN	RNA-RNA interactions: non-targeting interactions	miR-125b-5p (and other miRNAs w/seed CCCUGAG)
JUN	expression	IL10
JUN	expression	JUN
JUN	protein-DNA interactions	JUN
JUN	protein-DNA interactions	TGFB1
JUN	regulation of binding	JUN
PIK3R1	expression	PIK3R1
TGFB1	activation	JUN
TGFB1	expression	CD40
TGFB1	expression	JAG1
TGFB1	expression	JUN
TGFB1	expression	TGFB1
TGFB1	expression	TGFBR1
TGFB1	expression	TGFBR2
TGFB1	expression	let-7a-5p (and other miRNAs w/seed GAGGUAG)
TGFB1	expression	miR-125b-2-3p (and other miRNAs w/seed CAAGUCA)
TGFB1	expression	miR-17-5p (and other miRNAs w/seed AAAGUGC)
TGFB1	expression	miR-181a-5p (and other miRNAs w/seed ACAUUCA)

TGFB1	expression	miR-18a-5p (and other miRNAs w/seed AAGGUGC)
TGFB1	localization	TGFB1
TGFB1	localization	TGFBR1
TGFB1	phosphorylation	JUN
TGFB1	phosphorylation	TGFBR1
TGFB1	protein-protein interactions	TGFBR2
TGFB1	regulation of binding	TGFBR1
TGFB1	transcription	JUN
TGFBR1	protein-protein interactions	JAK1
TGFBR1	protein-protein interactions	TGFBR2
TGFBR1	regulation of binding	TGFBR1
TGFBR2	expression	JUN
TGFBR2	expression	TGFB1
TGFBR2	expression	TGFBR1
TGFBR2	expression	TGFBR2
TGFBR2	phosphorylation	TGFB1
TGFBR2	phosphorylation	TGFBR1
TGFBR2	protein-protein interactions	TGFB1
TGFBR2	protein-protein interactions	TGFBR1
miR-128-3p (and other miRNAs w/seed CACAGUG)	expression	TGFBR1
miR-141-3p (and other miRNAs w/seed AACACUG)	expression	JAG1
miR-146a-5p (and other miRNAs w/seed GAGAACU)	RNA-RNA interactions: microRNA targeting	CCR3
miR-146a-5p (and other miRNAs w/seed GAGAACU)	RNA-RNA interactions: microRNA targeting	CD40
miR-146a-5p (and other miRNAs w/seed GAGAACU)	RNA-RNA interactions: microRNA targeting	IL10
miR-146a-5p (and other miRNAs w/seed GAGAACU)	RNA-RNA interactions: microRNA targeting	IL12RB2
miR-146a-5p (and other miRNAs w/seed GAGAACU)	activation	JUN
miR-146a-5p (and other miRNAs w/seed GAGAACU)	expression	CCR3
miR-146a-5p (and other miRNAs w/seed GAGAACU)	expression	CD40
miR-146a-5p (and other miRNAs w/seed GAGAACU)	expression	IL10

miR-146a-5p (and other miRNAs w/seed GAGAACU)	expression	IL12RB2
miR-146a-5p (and other miRNAs w/seed GAGAACU)	expression	TBX21
miR-146a-5p (and other miRNAs w/seed GAGAACU)	phosphorylation	JUN
miR-17-5p (and other miRNAs w/seed AAAGUGC)	RNA-RNA interactions: microRNA targeting	TGFBR2
miR-17-5p (and other miRNAs w/seed AAAGUGC)	expression	TGFBR2
miR-200b-3p (and other miRNAs w/seed AAUACUG)	expression	JAG1
miR-221-3p (and other miRNAs w/seed GCUACAU)	RNA-RNA interactions: microRNA targeting	PIK3R1
miR-221-3p (and other miRNAs w/seed GCUACAU)	expression	PIK3R1
miR-291a-3p (and other miRNAs w/seed AAGUGCU)	expression	TGFBR2
miR-495-3p (and other miRNAs w/seed AACAAAC)	expression	TGFBR2
miR-543-3p (and other miRNAs w/seed AACAUUC)	expression	TGFBR2

Table S7: **Relationships between the molecules (miRNAs and mRNAs) within the Th17 pathway**

From Molecule(s)	Relationship Type	To Molecule(s)
Hsp90	expression	Hsp90
IL10	activation	NFkB (complex)
IL10	activation	STAT3
IL10	expression	IL10
IL10	expression	IL17A
IL10	expression	IL1B
IL10	expression	IL6
IL10	localization	IL1B
IL10	localization	IL6
IL10	localization	RELA
IL10	phosphorylation	STAT3
IL10	regulation of binding	RELA
IL10	transcription	IL10
IL17A	activation	JAK1
IL17A	activation	NFkB (complex)

IL17A	activation	RELA
IL17A	expression	CCL20
IL17A	expression	IL1B
IL17A	expression	IL6
IL17A	expression	miR-129-5p (and other miRNAs w/seed UUUUUGC)
IL17A	localization	CCL20
IL17A	localization	IL10
IL17A	localization	IL1B
IL17A	localization	IL6
IL17A	phosphorylation	JAK1
IL17A	phosphorylation	STAT3
IL17A	translocation	RELA
IL1B	activation	JAK1
IL1B	activation	NFkB (complex)
IL1B	activation	RELA
IL1B	expression	CCL20
IL1B	expression	IL10
IL1B	expression	IL17A
IL1B	expression	IL1B
IL1B	expression	IL6
IL1B	localization	IL6
IL1B	modification	RELA
IL1B	molecular cleavage	TRAF6
IL1B	phosphorylation	JAK1
IL1B	phosphorylation	RELA
IL1B	phosphorylation	STAT3
IL1B	regulation of binding	CCL20
IL1B	regulation of binding	IL1B
IL1B	regulation of binding	NFkB (complex)
IL1B	regulation of binding	RELA
IL1B	regulation of binding	TRAF6
IL1B	transcription	CCL20
IL1B	transcription	IL1B
IL1B	transcription	IL6

IL1B	translocation	NFkB (complex)
IL1B	translocation	RELA
IL1R1	activation	NFkB (complex)
IL1R1	expression	IL1B
IL1R1	protein-protein interactions	IL1B
IL1R1	protein-protein interactions	TRAF6
IL1R1	regulation of binding	IL1B
IL1R1	regulation of binding	TRAF6
IL1R1	translocation	RELA
IL6	activation	JAK1
IL6	activation	STAT3
IL6	expression	IL17A
IL6	expression	IL6
IL6	expression	STAT3
IL6	localization	STAT3
IL6	phosphorylation	JAK1
IL6	phosphorylation	STAT3
IL6	regulation of binding	JAK1
IL6	regulation of binding	RELA
IL6	regulation of binding	STAT3
IL6	transcription	IL6
IL6	translocation	STAT3
IRAK2	activation	NFkB (complex)
IRAK2	expression	IL1B
IRAK2	protein-protein interactions	IRAK2
JAK1	activation	STAT3
JAK1	expression	IL10
JAK1	localization	IL10
JAK1	localization	IL6
JAK1	phosphorylation	STAT3
JAK1	protein-protein interactions	Hsp90
JAK1	protein-protein interactions	STAT3
NFkB (complex)	expression	CCL20
NFkB (complex)	expression	IL10
NFkB (complex)	expression	IL1B

NFkB (complex)	expression	IL6
NFkB (complex)	expression	miR-146a-5p (and other miRNAs w/seed GAGAACU)
NFkB (complex)	localization	IL10
NFkB (complex)	localization	IL1B
NFkB (complex)	phosphorylation	RELA
NFkB (complex)	protein-DNA interactions	IL6
NFkB (complex)	protein-protein interactions	RELA
NFkB (complex)	regulation of binding	NFkB (complex)
NFkB (complex)	regulation of binding	RELA
NFkB (complex)	transcription	IL1B
NFkB (complex)	transcription	IL6
NFkB (complex)	translocation	RELA
RELA	activation	NFkB (complex)
RELA	expression	CCL20
RELA	expression	IL10
RELA	expression	IL1B
RELA	expression	IL6
RELA	expression	RELA
RELA	expression	miR-200b-3p (and other miRNAs w/seed AAUACUG)
RELA	localization	CCL20
RELA	localization	IL6
RELA	localization	STAT3
RELA	molecular cleavage	RELA
RELA	protein-DNA interactions	CCL20
RELA	protein-DNA interactions	IL10
RELA	protein-DNA interactions	IL1B
RELA	protein-DNA interactions	IL6
RELA	protein-protein interactions	NFkB (complex)
RELA	protein-protein interactions	STAT3
RELA	regulation of binding	NFkB (complex)
RELA	transcription	CCL20
RELA	transcription	IL6
RELA	transcription	NFkB (complex)
RELA	translocation	RELA

STAT3	activation	NFkB (complex)
STAT3	expression	CCL20
STAT3	expression	IL10
STAT3	expression	IL17A
STAT3	expression	IL1B
STAT3	expression	IL6
STAT3	expression	STAT3
STAT3	expression	miR-181a-5p (and other miRNAs w/seed ACAUUCA)
STAT3	localization	IL10
STAT3	localization	IL17A
STAT3	localization	IL6
STAT3	localization	RELA
STAT3	localization	STAT3
STAT3	phosphorylation	STAT3
STAT3	protein-DNA interactions	IL10
STAT3	protein-DNA interactions	IL6
STAT3	protein-DNA interactions	STAT3
STAT3	protein-protein interactions	Hsp90
STAT3	protein-protein interactions	JAK1
STAT3	protein-protein interactions	NFkB (complex)
STAT3	protein-protein interactions	RELA
STAT3	regulation of binding	STAT3
STAT3	transcription	STAT3
TRAF6	activation	NFkB (complex)
TRAF6	activation	RELA
TRAF6	expression	IL1B
TRAF6	expression	IL6
TRAF6	localization	IL6
TRAF6	phosphorylation	RELA
TRAF6	protein-protein interactions	IL1R1
TRAF6	protein-protein interactions	IRAK2
TRAF6	transcription	IL10
TRAF6	transcription	RELA
TRAF6	translocation	RELA

miR-146a-5p (and other miRNAs w/seed GAGAACU)	RNA-RNA interactions: microRNA targeting	IL10
miR-146a-5p (and other miRNAs w/seed GAGAACU)	RNA-RNA interactions: microRNA targeting	IL12RB2
miR-146a-5p (and other miRNAs w/seed GAGAACU)	RNA-RNA interactions: microRNA targeting	IL1R1
miR-146a-5p (and other miRNAs w/seed GAGAACU)	RNA-RNA interactions: microRNA targeting	TRAF6
miR-146a-5p (and other miRNAs w/seed GAGAACU)	activation	NFkB (complex)
miR-146a-5p (and other miRNAs w/seed GAGAACU)	expression	IL10
miR-146a-5p (and other miRNAs w/seed GAGAACU)	expression	IL12RB2
miR-146a-5p (and other miRNAs w/seed GAGAACU)	expression	IL1R1
miR-146a-5p (and other miRNAs w/seed GAGAACU)	expression	RELA
miR-146a-5p (and other miRNAs w/seed GAGAACU)	expression	TRAF6
miR-31-5p (and other miRNAs w/seed GGCAAGA)	expression	IL1B
miR-491-5p (and other miRNAs w/seed GUGGGGA)	inhibition	Hsp90
miR-491-5p (and other miRNAs w/seed GUGGGGA)	inhibition	NFkB (complex)
miR-495-3p (and other miRNAs w/seed AACAAAC)	expression	IL6
miR-515-3p (and other miRNAs w/seed AGUGCCU)	activation	NFkB (complex)
miR-543-3p (and other miRNAs w/seed AACAUUC)	expression	IL6

Table S8: Upregulated miR-4633-5p-target genes in cancers

**miRNA**	**miR-4633-5p**	
**Target genes**	**Cancer Type**	**Reference**
hsa-miR-4633-5p (40 folds)	Potential Cancer-Related Urinary-miRNA	Yasui, doi: 10.1126/sciadv.1701133
PDZD11	Prostate Cancer	Xu, doi: 10.1186/s12967-018-1637-x
BCL2	Lung Cancer	Han, doi: 10.1016/j.ccell.2015.04.010
	Colon Cancer	Berger, doi: 10.7554/eLife.20352
Cyclin G1	Breast Cancer	Tian, doi: 10.1590/1414-431X20175612
	Ovarian Cancer	Liu, doi: 10.12659/MSM.895562
RAS	Pancreatic and Lung Cancer	O’Bryan, doi: 10.1016/j.phrs.2018.10.021
p21	Brain, lung, and colon cancer	Shamloo, doi: 10.3390/cancers11081178
A Kinase Anchor Protein 5	Stomach Adenocarcinoma	Zhong, doi: 10.21037/atm.2019.12.98
MDM4	Breast Cancer	Guo, doi: 10.18632/oncotarget.7533
Lim Domain	Head and Neck Cancer	Swetzig, doi: 10.1371/journal.pone.0164804

	Prostate Cancer	Simonik, doi: 10.18632/oncotarget.8359
	Breast Cancer	Jiang, doi: 10.1038/onc.2014.64
Integrin α2	Head and Neck Cancer	Liu, X, doi: 10.1038/srep23545
	Prostate Cancer	Yamada, doi: 10.1186/1476-4598-13-208
	Cervical Cancer	Ziaee, doi: 10.18632/oncotarget.6840
	Ovarian Cancer	Liu, Fei, doi: 10.1186/1471-2407-13-80
	Liver Cancer	Liu, Hao, doi: 10.18632/oncotarget.12760
	Colorectal Cancer	Wong, K, doi: 10.1158/0008-5472.CAN-09-0315
TMED	Breast Cancer	Yoshimura, doi: 10.2147/CMAR.S192949
	Breast and Ovarian Cancer	Lin, X, doi: 10.2147/CMAR.S278570
IL1A	Ovarian Cancer	Ge, Xueling, doi: 10.1158/0008-5472.CAN-13-1051
	Colorectal Cancer	Charbonneau, doi: 10.1186/s12885-019-5395-9
NR5A2	Lung Cancer	Ji, H, doi: 10.1002/cam4.1992
	Gastric Cancer	Ye, Ting, doi: 10.2147/OTT.S201228
	Pancreatic Cancer	Luo Z, doi: 10.1002/mc.22604
AK7	Ovarian Cancer	Zhang, X. Y., doi: 10.1097/MD.0000000000024134
FOXO3	Breast Cancer	Mahmud, doi: 10.3390/cancers11081067<br>Zhang, L., doi: 10.1172/JCI90077<br>Gong, C., doi: 10.1038/oncsis.2016.23
HLA-A	Esophageal and Gastric Cancer	Mimura, K, doi: 10.4049/jimmunol.1301597
NRIP1	Stomach Adenocarcinoma	Fang, D., doi: 10.21037/atm-20-6197
	Breast Cancer	Aziz, M. H, doi: 10.18632/oncotarget.5356<br>Lei, Jin-Ju: doi: 10.18632/oncotarget.4573
	Esophageal Squamous Cell Carcinoma	Chen, X, doi: 10.1186/s13046-020-01640-9
	Lymphocytic leukemia	Lapierre, M, doi: 10.1186/s13045-015-0116-6
	Gastric Cancer	Liu, Y., doi: 10.2147/CMAR.S245941
NFATC2IP		
CYP2B		
GPR13		
Cadherin	Gastric, lung, pancreatic, ovarian, breast cancer, glioblastoma	Kourtidis, A, doi: 10.1016/j.yexcr.2017.04.006
AUKB		
Leptin	Breast and Gynecologic Cancer	Crean-Tate, doi: 10.1210/en.2018-00379
SRC (SKAP2)		
TNF	Lung Cancer	Gong, K, doi: 10.1172/JCI96148
	Prostate Cancer	Vickman, R. E., doi: 10.1158/1541-7786.MCR-18-1054
	Colorectal Cancer	Buhrmann, doi: 10.3390/nu11030704
ADAM22	Breast Cancer	Charmsaz, S., doi: 10.1186/s12916-020-01806-4
IL4R	Epithelial Cancer	Bankaitis, K. V., doi: 10.1007/s10585-015-9747-9
	Gastrointestinal Cancer	Cho, Y. A., doi: 10.1016/j.je.2016.06.002

Table S9: Patient demographic of TNM (T1) staging with age, sex, BMI with miR-4633-5p expression in Filipinos vs. Europeans

Patient No.	Age	Sex	Ethnicity	Type of thyroid cancer	pTNM	High BMI	miR-4633-5p
Pt#75	28	M	Asian (FA)	PTC	pT1aN1a (Stage III)	+	-5.36
Pt#89	45	F	Asian (FA)	PTC (microcarcinoma)	pT1aNX (Stage I)	+	-5.02
Pt#88	55	F	Asian (FA)	Follicular variant of PTC	pT1aNX (Stage I)	-	-2.89
Pt#87	24	F	Asian (FA)	Follicular variant of PTC	pT1aNX (Stage I)	+	-2.06

Pt#86	55	M	Asian (FA)	Follicular variant of PTC	pT1aNX (Stage I)	+	-1.72
Pt#85	36	F	Asian (FA)	PTC	pT1bN0 (Stage I)	+	-1.56
Pt#76	48	F	Asian (FA)	PTC	pT1aN1a (Stage II)	+	-1.26
Pt#77	49	F	Asian (FA)	PTC	pT1bN1b (Stage III)	+	-1.13
Pt#78	37	F	Asian (FA)	PTC	pT1aNX (Stage II)	+	-1.1
Pt#84	35	M	Asian (FA)	PTC	pT1aN0 (Stage I)	+	-1.05
Pt#83	74	M	Asian (FA)	Follicular variant of PTC	pT1aNx (Stage I)	-	-0.89
Pt#82	50	F	Asian (FA)	PTC	pT1a NX (Stage I)	+	0.84
Pt#43	33	F	White	PTC	pT1a N0 (Stage I)	-	-11.46
Pt#42	34	F	White	PTC	pT1a NX (Stage I)	-	-7.3
Pt#41	43	F	White	PTC	pT1aNX (Stage I)	-	-7.16
Pt#36	80	M	White	PTC	pT1a pN1a (Stage III)	-	-6.91
Pt#81	45	F	Asian (FA)	PTC	pT1b pN1a (Stage I)	+	-0.31
Pt#35	74	F	White	Follicular variant of PTC	pT1aNX (Stage II)	+	-6.78
Pt#40	69	F	White	Micropapillary carcinoma	pT1NX (Stage I)	-	-6.72
Pt#39	33	F	White	PTC	pT1a N0 (Stage I)	+	-6.48
Pt#38	49	F	White	PTC	pT1a N0 (Stage I)	-	-6.35
Pt#37	34	F	White	PTC	pT1a N1 (Stage I)	-	-6.24
Pt#80	43	F	Asian (FA)	PTC	pT1aNX (Stage I)	+	-0.29

Pt#34	80	M	White	PTC with microcalcification	pT1a pN1a (Stage III)	-	-6.12
Pt#79	45	F	Asian	PTC	pT1b pN1a (Stage II)	-	-0.27
Pt#33	74	F	White	PTC with calcification	pT1 NX (Stage I)	-	-5.34
Pt#32	75	M	White	PTC with microcalcification	pT1a(m) N1a MX (Stage III)	-	-5.33
Pt#74	45	M	Asian (FA)	Follicular variant of PTC	pT1aNX (Stage I)	+	0.55
Pt#73	35	F	Asian (FA)	PTC with microcalcification	pT1N1a (Stage II)	+	1.15
Pt#72	23	F	Asian (FA)	PTC with microcalcification	pT1bN1a (Stage I)	+	3.56
Pt#71	50	F	Asian (FA)	PTC	pT1a NX (Stage II)	+	5.32
Pt#31	59	F	White (EA)	Micropapillary carcinoma	pT1NX (Stage I)	-	-4.52
Pt#30	33	F	White (EA)	PTC	pT1a N0 (Stage I)	-	-4.42
Pt#29	49	F	White (EA)	PTC	pT1a N0 (Stage I)	-	-4.38
Pt#28	34	F	White (EA)	PTC	pT1a N1 (Stage II)	+	-4.14
Pt#70	43	F	Asian (FA)	PTC	pT1aNX (Stage I)	+	5.96
Pt#69	45	F	Asian (FA)	PTC	pT1b pN1a (Stage II)	-	5.98
Pt#68	35	F	Asian (FA)	PTC	pT1aN0 (Stage I)	-	
Pt#27	74	F	White (EA)	Follicular variant of PTC	pT1aNX (Stage I)	+	-3.65
Pt#67	50	F	Asian (FA)	PTC	pT1a NX (Stage I)	+	6.22
Pt#26	75	M	White (EA)	PTC with microcalcification	pT1a(m) N1a MX (Stage II)	+	-2.66

Pt#25	33	F	White (EA)	PTC	pT1a N0 (Stage I)	-	-2.59
Pt#24	49	F	White (EA)	PTC	pT1a NX (Stage I)	-	-2.39
Pt#66	27	F	Asian (FA)	PTC with microcalcification	pT1 NX (Stage I)	+	6.38
Pt#23	34	F	White (EA)	PTC	pT1a NX (Stage I)	+	-2.03
Pt#22	43	F	White (EA)	PTC	pT1NX (Stage I)	+	-1.56
Pt#65	45	F	Asian (FA)	PTC	pT1b pN1a (Stage II)	+	6.83
Pt#64	25	F	Asian (FA)	PTC	pT1aN0 (Stage I)	+	6.99
Pt#21	74	F	White (EA)	Follicular variant of PTC with calcification	pT1aNX (Stage I)	-	-1.37
Pt#20	24	F	White (EA)	Follicular carcinoma	pT1N0 (Stage I)	-	-0.23
Pt#63	50	F	Asian (FA)	PTC	pT1a NX (Stage I)	+	7.48
Pt#19	75	M	White (EA)	PTC with calcification	pT1a(m) N1a Mx (Stage III)	+	0.14

Pt#, patient number; F, female; M, male; p, pathological; T, tumor size (T1a Tumor 1 cm or less, limited to the thyroid; T1b: Tumor more than 1 cm but not more than 2 cm in greatest dimension, limited to the thyroid; T2; Tumor more than 2 cm but not more than 4 cm in greatest dimension, limited to the thyroid; T3: Tumor more than 4 cm in greatest dimension, limited to the thyroid or any tumor with minimal extrathyroid extension (e.g., extension to sternothyroid muscle or perithyroid soft tissues); N, Regional node; NX, Regional lymph nodes cannot be assessed; N0, No regional lymph node metastasis; N1, Regional lymph node metastasis; N1a, Metastasis to Level VI (pretracheal, paratracheal, and prelaryngeal lymph nodes); N1b, Metastasis to unilateral, bilateral, or contralateral cervical Levels I, II, III, IV, or V) or superior mediastinal lymph nodes (Level VII) M, metastasis; M0, No distant metastasis; M1, Distant metastasis. +, yes; -, no. PTC, papillary thyroid cancer; FA, Filipino Americans; EA, Europeans Americans; M, male; F, female

Table S10: Patient demographic of TNM (T2) staging with age, sex, BMI with miR-4633-5p expression in Filipinos vs. Europeans

Patient No.	Age	Sex	Ethnicity	Type of thyroid cancer	pTNM	High BMI	miR-4633-5p
Pt#62	25	F	Asian (FA)	PTC	pT2N1a (Stage II)	+	7.58
Pt#61	36	F	Asian (FA)	PTC with microcalcification	pT2N1a (Stage II)	-	8.21

Pt#60	49	F	Asian (FA)	PTC with microcalcification	pT2NX (Stage II)	+	8.42
Pt#59	79	F	Asian (FA)	Follicular variant of papillary carcinoma microcalcification	pT2N0 (Stage III)	+	8.73
Pt#58	20	F	Asian (FA)	PTC with calcification	pT2NX (Stage II)	+	8.81
Pt#18	59	M	White (EA)	Micropapillary carcinoma with calcification	pT2N1a (Stage III)	+	0.62
Pt#17	49	F	White (EA)	PTC with microcalcification	pT2a N1 (Stage III)	+	1.23
Pt#57	77	F	Asian (FA)	PTC	pT2 Nx (Stage II)	-	9.17
Pt#16	26	F	White (EA)	PTC with microcalcifcation	pT2NX (Stage I)	+	1.7
Pt#56	65	M	Asian (FA)	PTC	pT2N0 (Stage II)	+	10.05
Pt#55	20	F	Asian (FA)	PTC	pT2NX (Stage I)	-	10.85
Pt#54	77	F	Asian (FA)	PTC with microcalcification	pT2 NX (Stage II)	+	11.1
Pt#15	74	F	White (EA)	PTC	pT2 NX (Stage II)	-	2.42
Pt#53	20	F	Asian (FA)	PTC with microcalcification	pT2NX (Stage I)	+	11.29
Pt#14	59	F	White (EA)	Micropapillary carcinoma with microcalcification	pT2N1 (Stage III)	+	3.65
Pt#13	26	F	White (EA)	PTC	pT2NX (Stage I)	-	4.32
Pt#12	74	F	White	PTC	pT2 NX (Stage II)	-	4.84
Pt#52	20	F	Asian (FA)	PTC with microcalcification	pT2NX (Stage I)	+	12.24
Pt#51	77	F	Asian (FA)	PTC	pT2 NX (Stage II)	+	13.67

Pt#11	34	F	White (EA)	PTC	pT2a N1 (Stage I)	-	5.4
Pt#10	26	F	White (EA)	PTC	pT2NX (Stage I)	-	5.52
Pt#9	43	F	White (EA)	PTC	pT2NX (Stage I)	+	5.73
Pt#8	74	F	White (EA)	PTC	pT2 NX (Stage II)	-	6.18

Pt#, patient number; F, female; M, male; p, pathological; T, tumor size (T1a Tumor 1 cm or less, limited to the thyroid; T1b: Tumor more than 1 cm but not more than 2 cm in greatest dimension, limited to the thyroid; T2; Tumor more than 2 cm but not more than 4 cm in greatest dimension, limited to the thyroid; T3: Tumor more than 4 cm in greatest dimension, limited to the thyroid or any tumor with minimal extrathyroid extension (e.g., extension to sternothyroid muscle or perithyroid soft tissues); N, Regional node; NX, regional lymph nodes cannot be assessed; N0, no regional lymph node metastasis; N1, regional lymph node metastasis; N1a, Metastasis to Level VI (pretracheal, paratracheal, and prelaryngeal lymph nodes); N1b, Metastasis to unilateral, bilateral, or contralateral cervical Levels I, II, III, IV, or V) or superior mediastinal lymph nodes (Level VII) M, metastasis; M0, No distant metastasis; M1, Distant metastasis. +, yes; -, no. DBP, vitamin D binding protein; PTC, papillary thyroid cancer; FA, Filipino Americans; EA, Europeans Americans; M, male; F, female.

Table S11: Correlation of clinicopathological features with TNM (T3) staging age, sex, with miR-4633-5p expression in Filipinos vs. European

Patient No.	Age	Sex	Ethnicity	Types of thyroid cancer	pTNM	High BMI	miR-4633-5p
Pt#44	72	F	Asian (FA)	PTC with Follicular variant	pT3N1b (stage IV)	+	13.86
Pt#45	70	M	Asian (FA)	PTC with microcalcification	pT4aN1b (Stage IV)	-	13.95
Pt#46	40	F	Asian (FA)	PTC/ATC microcalcification	pT4aN1b (Stage IV)	+	14.04
Pt#47	25	F	Asian (FA)	PTC with Follicular variant with calcification	pT3N1a (Stage I)	+	14.23
Pt#48	66	M	Asian (FA)	PTC with Follicular variant with calcification	pT3N1a (Stage III)	-	14.56
Pt#49	34	F	Asian (FA)	PTC with Follicular variant with calcification	pT3 N1 (Stage III)	+	14.62
Pt#50	24	F	Asian (FA)	PTC with Follicular variant with calcification	pT3N1a (Stage III)	+	NA
Pt#7	89	M	White (EA)	PTC with microcalcification	pT3 (m) pN1a (Stage III)	-	6.31

Pt#	46	M	Asian (FA)	PTC with calcification	pT3N1a (Stage II)	+	N/A
Pt#6	84	F	White (EA)	PTC with Follicular variant	pT3 N1 (Stage III)	-	6.84
Pt#5	74	F	White (EA)	PTC with Follicular variant	pT3N1a (Stage III)	-	7.03
Pt#4	66	F	White (EA)	PTC	pT3N1 (Stage III)	+	7.08
Pt#	32	F	Asian (FA)	PTC	pT3N1a (Stage III)	+	N/A
Pt#	84	F	Asian (FA)	PTC with Follicular variant	pT3N1a (Stage III)	-	N/A
Pt#	47	F	Asian (FA)	PTC with microcalcification	pT3N1aM0 (Stage III)	+	N/A
Pt#	42	F	Asian (FA)	PTC with calcification	pT3 (m) pN1a (Stage III)	+	N/A
Pt#	76	M	Asian (FA)	PTC with microcalcification	pT3N1a (Stage III)	-	N/A
Pt#3	74	F	White (EA)	PTC with microcalcification	pT3 N1 (Stage III)	-	7.5
Pt#	24	F	Asian (FA)	PTC microcalcification	pT3N1a (Stage III)	+	N/A
Pt#	42	F	Asian (FA)	PTC with microcalcification	pT3 (m) pN1a (Stage III)	+	N/A
Pt#	40	F	Asian (FA)	PTC with calcification	pT3N1a (Stage I)	-	N/A
Pt#	51	F	Asian (FA)	PTC with microcalcification	pT3N1a (Stage III)	+	N/A
Pt#	86	M	Asian (FA)	PTC with microcalcification	pT3N1 (Stage III)	-	N/A
Pt#	24	F	Asian (FA)	PTC with Follicular variant with calcification	pT3 N1 (Stage I)	+	N/A
Pt#96	42	F	Asian (FA)	PTC with microcalcification	pT3 (m) N1a (Stage II)	+	N/A

Pt#, patient number; F, female; M, male; p, pathological; T, tumor size (T1a Tumor 1 cm or less, limited to the thyroid; T1b: Tumor more than 1 cm but not more than 2 cm in greatest dimension, limited to the thyroid; T2; Tumor more than 2 cm but not more than 4 cm in greatest dimension, limited to the

thyroid; T3: Tumor more than 4 cm in greatest dimension, limited to the thyroid or any tumor with minimal extrathyroid extension (e.g., extension to sternothyroid muscle or perithyroid soft tissues); T4 All anaplastic carcinoma are considered T4 tumors; N, Regional node; NX, Regional lymph nodes cannot be assessed; N0, No regional lymph node metastasis; N1, Regional lymph node metastasis; N1a, Metastasis to Level VI (pretracheal, paratracheal, and prelaryngeal lymph nodes); N1b, Metastasis to unilateral, bilateral, or contralateral cervical Levels I, II, III, IV, or V) or superior mediastinal lymph nodes (Level VII) M, metastasis; M0, No distant metastasis; M1, Distant metastasis; FA, Filipino Americans; EA, Europeans Americans; M, male; F, female; N/A, not available (not done).

Table S12: Downregulated miR-491-5p-target genes in cancers

**miRNA**	**miR-491-5p**	
**Target genes**	**Cancer type**	**Reference**
SHISA6	Gastric Cancer	Hao, Shuhong, DOI: 10.12659/MSM.915382
FOXP4	Prostate Cancer	Long, Qing-Zhi. https://doi.org/10.1371/journal.pone.0037866
	Bladder Cancer	Liang, Haote. https://doi.org/10.1042/BSR20193484
DERL3	Gastric Cancer	Li, Yongtuan. https://doi.org/10.1016/j.compbiolchem.2019.107172
FBXO41	Squamous-Cell Lung Carcinoma	Wang, K. https://doi.org/10.1007/s00432-018-2653-1
ZNF703	Breast Cancer	Sircuolomb, Fabrice. https://doi.org/10.1002/emmm.201100121
	Gastric Cancer	Yang, Gongli. https://doi.org/10.3892/or.2014.2997
	Head and Neck Squamous Cell Carcinoma	Orhan, Ceren. https://doi.org/10.1111/coa.13450

Table S13: Upregulated let-7 family-target genes in cancers

**miRNA**	**let-7a-2-3p/let-7g-3p**	
**Target genes**	**Cancer Type**	**Reference**
DCUN1D1	Cervical Cancer	Jiang, Zhaojin. doi: 10.18632/oncotarget.9850
	Squamous Cell Carcinoma	Sarkaria, Inderpal. DOI: 10.1158/0008-5472.CAN-06-2074 Published October 2006
	Prostate Cancer	Zhang, Z. H. https://pubmed.ncbi.nlm.nih.gov/29077169/
TBP	Ovarian Cancer	Ribeiro, Jennifer. https://doi.org/10.3389/fonc.2014.00045
	Bladder Cancer	Ohl, Falk. https://doi.org/10.1016/S0022-5347(05)00919-5
IGBP1	Breast Cancer	Chen, D. https://doi.org/10.1186/s12935-020-01214-x
C1QBP	Prostate Cancer	Amamoto, Rie. https://doi.org/10.1111/j.1349-7006.2010.01828.x
	Colon Cancer	Kim, Kun. https://doi.org/10.3892/etm.2017.4249
	Pancreatic Cancer	Haojun, Shi. https://doi.org/10.1002/ijc.30831
**miRNA**	**let-7i-3p**	
**Target genes**		
DLX5	Breast Cancer	Morini, M. https://doi.org/10.1186/1471-2407-10-649
	Ovarian Cancer	Tan, Yinfei. DOI: 10.1158/0008-5472.CAN-10-1568
	Non-Small Cell Lung Cancer	Sun, Shanshan. https://doi.org/10.1016/j.lfs.2020.118508
HRH2	Gastric Cancer	Arisawa, Tomiyasu. https://doi.org/10.3892/ijo.2012.1482
CDR2	Colorectal Cancer	Mo, JS. https://doi.org/10.1007/s13258-020-01016-5
CBX7	Thyroid Cancer	Pallante, Pierlorenzo. DOI: 10.1158/0008-5472.CAN-08-0695
	Pancreatic Cancer	Karamitopoulou, Eva. https://doi.org/10.1016/j.ejca.2010.01.033
	Gastric Cancer	Pallante, Pierlorenzo. https://doi.org/10.1371/journal.pone.0098295
